# Prediction of the water solubility by a graph convolutional-based neural network on a highly curated dataset

**DOI:** 10.1186/s13321-025-01000-9

**Published:** 2025-04-21

**Authors:** Nadin Ulrich, Karsten Voigt, Anton Kudria, Alexander Böhme, Ralf-Uwe Ebert

**Affiliations:** 1 Department of Exposure Science, Helmholtz Centre for Environmental Research-UFZ, Permoserstrasse 15, 04318 Leipzig, Germany; 2 PAULY, Theresienstrasse 50, 04129 Leipzig, Germany

**Keywords:** Water solubility, Neural networks, Machine learning, Physico-chemical property prediction

## Abstract

Water solubility is a relevant physico-chemcial property in environmental chemistry, toxicology, and drug design. Although the water solubility is besides the octanol–water partition coefficient, melting point, and boiling point a property with a large amount of available experimental data, there are still more compounds in the chemical universe for which information on their water solubility is lacking. Thus, prediction tools with a broad application domain are needed to fill the corresponding data gaps. To this end, we developed a graph convolutional neural network model (GNN) to predict the water solubility in the form of log *S*_w_ based on a highly curated dataset of 9800 chemicals. We started our model development with a curation workflow of the AqSolDB data, ending with 7605 data points. We added 2195 chemicals with experimental data, which we found in the literature, to our dataset. In the final dataset, log *S*_w_ values range from − 13.17 to 0.50. Higher values were excluded by a cut-off introduced to eliminate fully miscible chemicals. We developed a consensus GNN by a fivefold split of the corresponding training set (70% of the data) and validation set (20%) and used 10% as independent test set for the evaluation of the performance of the different splits and the consensus model. By doing so, we achieved an *r*^2^ of 0.901, a *q*^2^ of 0.896, and an *rmse* of 0.657 on our independently selected test set, which is close to the experimental error of 0.5 to 0.6 log units. We further provide the information on the application domain and compare our performance to other existing prediction tools.

**Scientific contribution** Based on a highly curated dataset, we developed a neural network to predict the water solubility of chemicals for a broad application domain. Data curation was done by us in a step-wise procedure, where we identified various errors in the experimental data. Based on an independent test set, we compare our prediction results to those of the available prediction models.

**Supplementary Information:**

The online version contains supplementary material available at 10.1186/s13321-025-01000-9.

## Introduction

Knowledge on water solubility is relevant in the fields of environmental chemistry and risk assessment of chemicals, where it determines the transport and fate of chemicals in the environment [1, 2]. Further, it plays a significant role in pharmacology and toxicology, especially in the ADME processes (absorption, distribution, metabolism, and excretion), and is, therefore, a main characteristic of drug design [3–6]. When it comes to effect concentrations in toxicology, the water solubility impacts the freely dissolved concentration of a chemical [7, 8]. Concentrations above the water solubility may lead to precipitation of the corresponding chemicals, which should, of course, be avoided in test systems like in-vitro assays for example.

The water solubility is defined as the maximal amount of a chemical (i.e., the solute) that can be dissolved in a defined volume of water. Water solubility *S*_w_ (standard units are mol/L or g/L) is often given in logarithmic form log *S*_w_. There are different definitions of water solubility: intrinsic water solubility refers to the solubility of the neutral chemical in water, whereas apparent water solubility refers to the water solubility at a certain pH, which is important for ionizable chemicals [9].

Temperature, ambient pressure, and pH level can be the major drivers of the solubility of chemicals in aqueous media. The main experimental methods for determining the water solubility are the saturation shake-flask method [10], the column elution/generator column method [10], the dissolution titration template method [3, 11, 12], and direct UV measurements [3, 13, 14].

One of the largest datasets which is freely available is the AqSolDB [15], which is a compilation of water solubility data collected from the eChemportal [16], EPI Suite [17], and the datasets of Raevsky et al. [18], Huuskonen [19], Wang et al. [20], Delaney [21], and Llinas et al. [22]. A broad overview on the different solubility datasets and models related to them is given by Llompart et al. [23]. Although the collection of physico-chemical data and, therefore, the number of models being developed based on these datasets is rising, there is evidence that curation of the datasets might be an option to improve the performance of the models [15, 24]. Especially the pH-dependency of the water solubility of a corresponding chemical, the formation of micelles, and the effects of co-solvents might have a substantial impact on the quality of data and, therefore, on the performance of the models [3].

Based on the AqSolDB, Sorkun et al. developed a consensus machine learning approach applying a set of chemical descriptors (atom-based, ring-based, bond-based, log *P*, topological, and E-state indices) with an overall *rmse* of 0.53 based on a test set of 1290 chemicals [25]. They used the different subsets of their initial data collection to develop various models and to combine them in a consensus approach. By doing so, they observed a direct relation between size of the dataset & data quality and the accuracy of the model. A different approach was used by Tang et al. [26]. The authors developed a self-attention-based message-passing neural network to predict log *S*_w_ with an overall *rmse* of 0.66 based on a dataset of 1311 chemicals. The main advantage of this approach is that heatmaps of the corresponding structures are generated, which highlight certain areas impacting log *S*_w_. However, the dataset used for the development of the neural network is relatively small. A multiple linear regression model (descriptors clogP, molecular weight, rotatable bonds, and aromatic proportion) was developed by Delaney based on a set of 2874 chemicals [21]. The model’s performance was evaluated on a blind test set of 528 chemicals, with a corresponding standard error of 0.96 [21]. The Delaney dataset is sometimes used as a benchmark in the literature [26, 27]. Tang et al. achieved an *rmse* of 0.66 on a subset of this set of chemicals (1311 chemicals), which is also used by Wu et al. (*rmse* = 0.58 (1128 chemicals), *rmse* = 0.68 for the subset of 1311 chemicals) for the evaluation of the performance. Tetko et al. developed a neural network applying the molecular weight and elecotrotopological E-state indices based on a set of 1291 chemicals, which achieved an overall *rmse* of 0.62 [28]. There are more models available, which were developed using different descriptors to predict log *S*_w_ values [19, 20]. However, Tang et al. indicated that graph-based neural networks tend to be more promising than conventional descriptor-based models [26]. They argue that the atoms represented as nodes being connected with bonds represented as edges, may depict the overall structure in a better way, which is in its 2D form more closely related to the corresponding property.

Thus, our aim was to develop a graph convolutional neural network (GNN) model for predicting log *S*_w_ for neutral chemicals based on a highly curated water solubility dataset. As most of the approaches used relatively small datasets for the model development, our aim was to develop the GNN on a larger dataset of experimental data. We used the AqSolDB dataset of Sorkun et al. [15] and developed a workflow to identify potential errors in the dataset. We preferred to use a data curation strategy as a first step, as it is known that not all datasets of log *S*_w_ values included high-quality data [15, 25]. Further, we extended the dataset with log *S*_w_ values of 2195 additional chemicals, which we collected from different sources. Based on the highly curated dataset of 9800 chemicals, we developed an consensus GNN model (based on a fivefold split into training and validation sets) to allow for a higher accuracy of log *S*_w_ predictions. We compare our results to the models of Sorkun et al. [25], Tang et al. [26] and the current model of Tetko et al. [28] (implemented in OCHEM [29]) as well as the two software tools ACD Galas [30] and EPI Suite [17]. Based on our prediction outcomes, we demonstrate that we can achieve an improved performance in the prediction of log *S*_w_ with our developed consensus GNN.

## Methods

### Solubility dataset

We used the AqSolDB dataset of Sorkun et al. [15] as a starting point for our model development. This dataset has already been curated with respect to chemical identifiers. The dataset is a comprehension of different solubility datasets from the literature. Thus, there are 7746 compounds with one log *S*_w_ value each and 2236 chemicals with multiple log *S*_w_ values. The corresponding log *S*_w_ values for chemicals with multiple values were selected based on the mean and the standard deviation. In the case of two different log *S*_w_ values for a chemical in the dataset, the deviation from a predicted reference value was used as a criterion. The merged repository contained 9982 chemicals in total. We removed inorganic chemicals, salts, mixtures, reactive chemicals, and polymers from this dataset. Finally, we ended up with 7605 chemicals.

The dataset was further extended by 2254 additional organic chemicals with their corresponding log *S*_w_ values, which we collected from the literature. However, due to the cut-off for large chemicals (molecular weight > 900 g/mol) and miscibility with water (log Sw > 0.5), the number was reduced to 2195 chemicals.

We developed some initial GNNs on the dataset to identify potential erroneous log *S*_w_ values. In the case of chemicals with multiple log *S*_w_ values given in the dataset, we selected the log *S*_w_ value, which was closer to the corresponding prediction. Additionally, we checked the corresponding literature or database for the original log *S*_w_ value and corrected the dataset. Further, we considered homologous series and similar structures in the dataset to indicate potential errors and issues.

### Neural networks

The curated dataset included 9800 chemicals. The dataset was randomly split into 70% training set, 20% validation set, and 10% test set. We checked that all structural features of the chemicals were homogeneously included in the three different sets. We first split off 10% of the test set and distributed the remaining chemicals along the training and validation set in five different setups. By this, we developed five independent sets, which were used for the fivefold cross-validation and as a basis for a consensus GNN.

To develop our GNN model, we first enlarged the number of input features by applying a data augmentation strategy. For data augmentation, different SMILES variants were generated using Openbabel (3.1.1), namely the canonical SMILES, the universal SMILES, and the inchified SMILES. All variants were generated with explicit hydrogens and with or without dative bonds. Further, the kekulized variant and the variant using aromatic labels were used. Based on these SMILES, all tautomeric forms were generated using RDKit version 2023.09.6. Duplicates of the SMILES variants were removed afterward. To avoid the over-weighting of chemicals with many tautomers, the number of tautomers was cut randomly to 50.

The development of the neural networks was done in Python version 3.11.8, Tensorflow version 2.15.0, and Keras version 2.15.0. The library Deepchem version 2.7.2 [31] was used to develop the GNNs. As input, molecular graphs were selected [27], which were generated from the SMILES of the corresponding chemicals using Deepchem (ConvMolFeaturizer). We selected molecular graphs, as the connectivity and the chemical bonding are represented, and we assumed that local interactions as well as the global structure (like shape and size) might be represented well by them. Both are of high relevance for the description of physico-chemical properties like log *S*_w_. We adapted the Keras implementation for the regression, the python code is provided at the GitHub repository. In brief, atoms and their corresponding properties (like atom type, implicit valence, hybridization, formal charge, aromaticity, chirality, e.g.) are represented as nodes, bonds to neighbor atoms are represented as edges. The graph convolutional operation involves the aggregation of the features of the neighboring atoms, in detail this is depicted by a graph convolutional layer, followed by a batch normalization and a graph pool layer. The output layer includes a dense and a batch normalization layer.

The calculations were performed on a Tuxedo book (Intel core i9, 64 GB RAM) with an NVIDIA RTX4090 (16 GB GDDR6). Additionally, the scientific results have, in part, been computed at the High-Performance Computing (HPC) Cluster EVE, a joint effort of both the Helmholtz Centre for Environmental Research—UFZ and the German Centre for Integrative Biodiversity Research (iDiv) Halle-Jena-Leipzig.

To identify the optimal setup (number of neurons, learning rate, activation function, loss function, number of epochs) for the training, neural networks with different setups were trained over 200 epochs, and the *rmse* (root mean squared error) was plotted over the epochs for both the training set and validation set (SI1). First, we tested different number of neurons per layer and different learning rates (Figure S1-1 and S1-2), second we tested different loss functions (Figure S1-3), and third we tested different activation functions (Figure S1-4) to identify the optimal structure and parameters. Based on these results, the final setup for our neural network was selected. Our GNN consists of two layers with 64 and 128 neurons; the learning rate is 0.0005, the dropout is set as 0.1, and the training is performed over 130 epochs. We applied a leaky ReLu function as an activation function and used the L1Loss function. Details on the code can be found in the GIT repository https://github.com/nadinulrich/log_Sw_prediction.

The applicability domain was determined according to Aniceto et al. [32], which combines the structure-based approach of Sahigara et al. [33] with the consensus standard deviation (reliability-based approach). In brief, the mean distance of each molecule in the training set to its k-nearest neighbors is determined, and the corresponding global reference value is calculated according to [32]. The mean Tanimoto distance is determined for each training set chemical to all neighbors within the radius of the reference value. The distances are further corrected according to the corresponding prediction reliability for each training set chemical. We, therefore, calculated the relative standard deviation of the prediction and the relative deviation from the experimental value, resulting in a correction factor for each radius as described in [32]. Details are provided in the github repository.

## Results and discussion

### Curation of the dataset

**Fig. 1:**
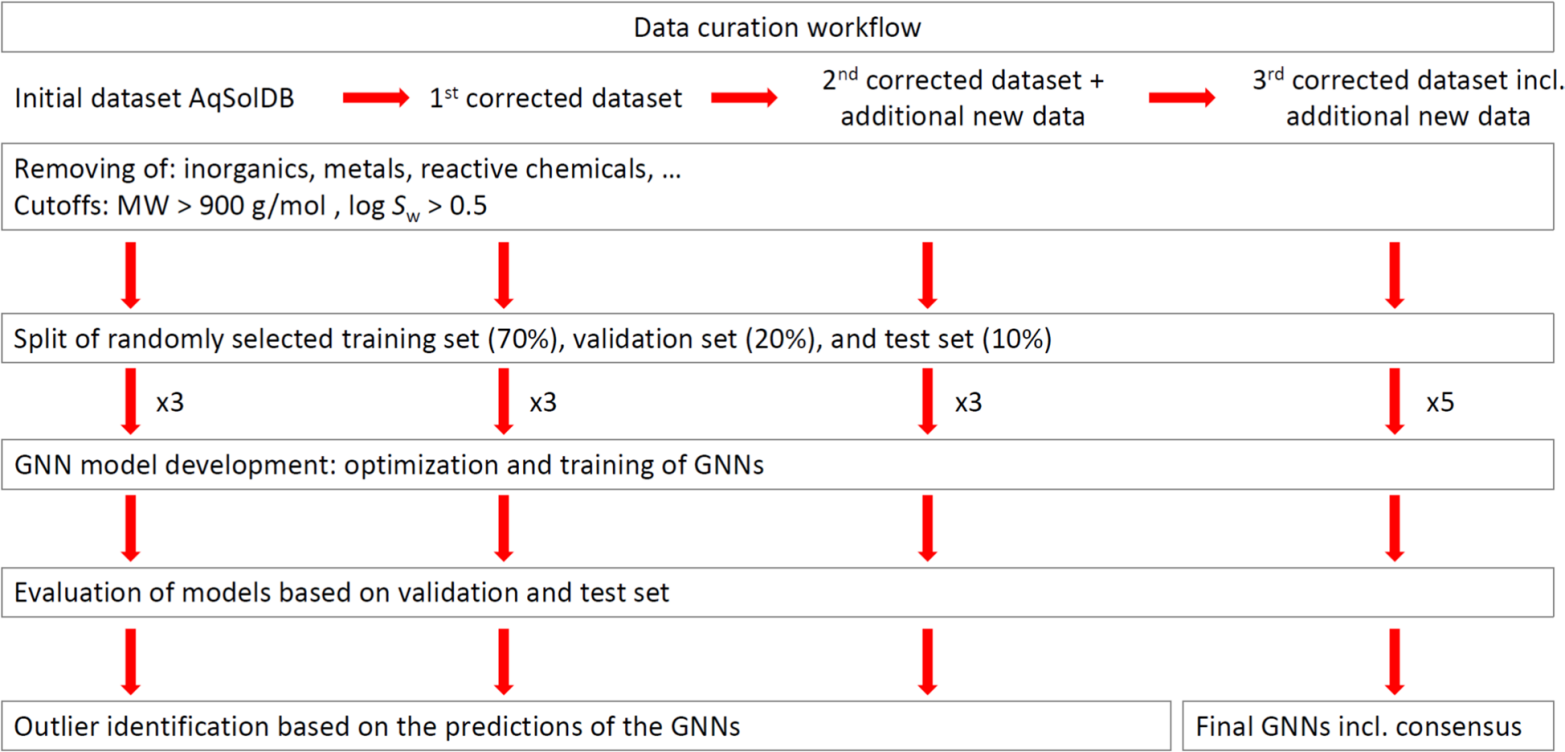
Flowchart to illustrate the data curation procedure

We started with the curation of the AqSolDB dataset published by Sorkun et al. [15] and performed an initial training of GNNs to identify potential outliers in the dataset (Fig. 1). The suggested solubility dataset of Sorkun et al. contained 9982 chemicals, comprised of a collection of several solubility datasets. Some chemicals were included in more than one dataset also with different log *S*_w_ values. For outliers (> 1 log unit difference to the experimental log *S*_w_) appearing in our initial training, we checked whether the corresponding chemical was included in several datasets and selected the corresponding log *S*_w_ value close to the corresponding prediction. We further checked the original sources in these cases to identify potential errors. The main errors found during our curation procedure were the following: (1) The log *S*_w_ value was given as “*below the limit of quantification*”, and the limit of quantification was included as log *S*_w_ in the dataset. In this case, we removed the value from the dataset, as the log *S*_w_ value might be orders of magnitude lower than the given value. (2) The value given in the dataset was a predicted value. This case often appeared in data from REACH dossiers in the ECHA database. Here, we removed the corresponding value. (3) Typos and errors resulting from data transfer; these data were corrected. (4) There were errors in the corresponding identifier of the chemical. If possible, we corrected the errors. Otherwise, we excluded the data from the dataset. (5) There were issues with the stability of the chemical in water, e.g., through hydrolysis; the corresponding data were excluded from the dataset. (6) In some cases, the critical micelle concentration was given instead of water solubility; these values were also excluded. We removed inorganic chemicals, complexes, and salts from the dataset and processed a check for duplicates. If multiple values were found in the original sources, we selected the log *S*_w_ value close to the predicted log *S*_w_ value. We are aware that this might lead to a bias, nevertheless in the case of multiple experimental values given for one chemical it is always hard to declare the “correct” one. Each correction is marked in the dataset provided in the GitHub repository. Further, we performed the screening for outliers three times based on the corresponding curated dataset. We included an additional cutoff for fully miscible chemicals; therefore, we excluded all log *S*_w_ values greater than 0.50. Additionally, we excluded chemicals with a molar mass > 900 g/mol. In total, 686 fully miscible chemicals were excluded, and 159 chemicals had molar mass > 900 g/mol.

**Fig. 2:**
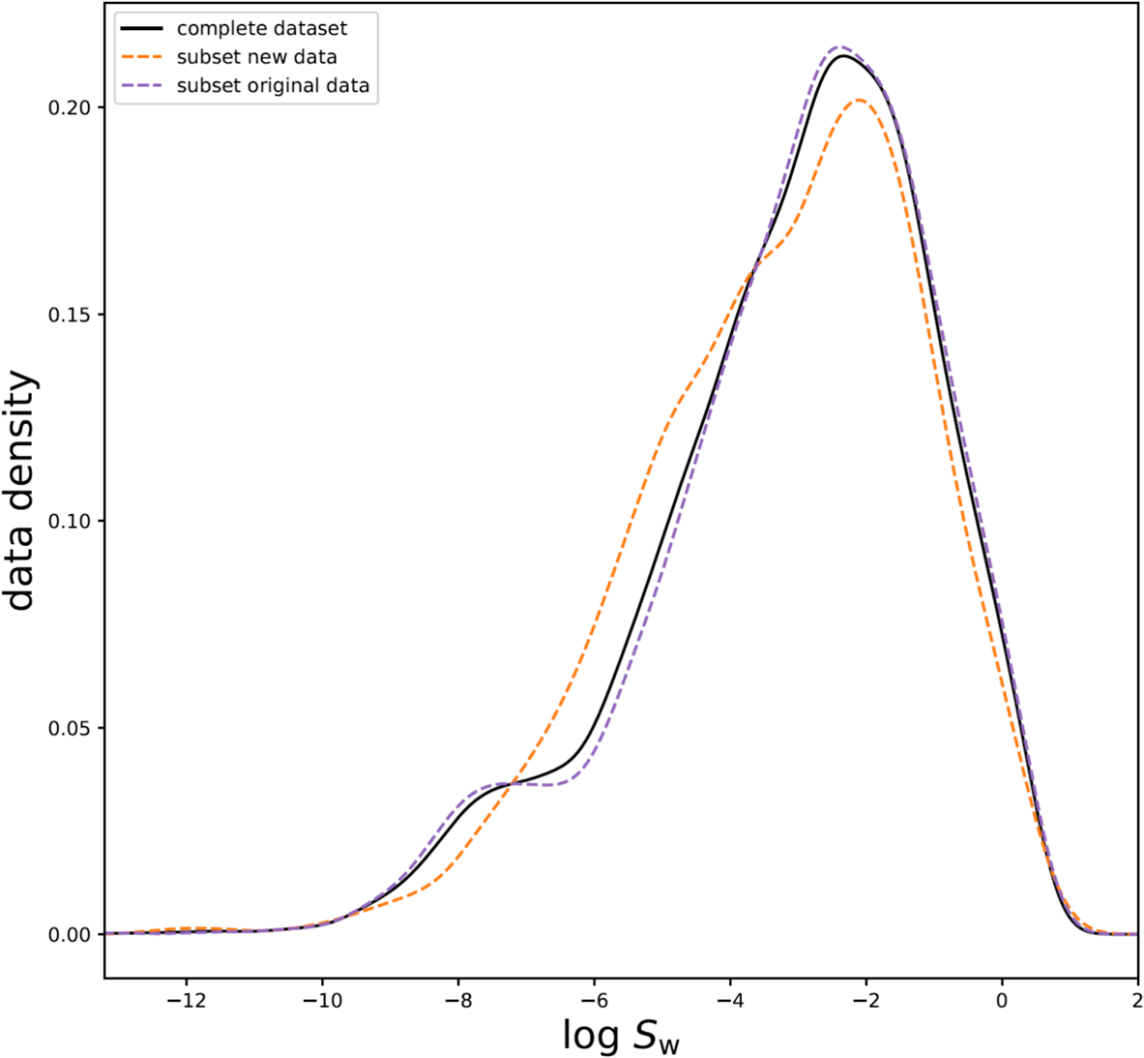
Density plot of the dataset used for model development. Density plot for the curated dataset. Besides the curated complete dataset, we added the information on the distribution of log *S*_w_ data for the subset of original data and the subset of new data (which we added to the original curated dataset)

The resulting dataset included log *S*_w_ values in the range of − 13.17 to 0.50. The distribution of all log *S*_w_ values in the dataset is depicted in the density plot for the curated complete dataset, the subset of original data, and the additional subset of new data (Fig. 2).

### Selection of the optimal GNN structure and performance of the GNN

The curated dataset contained log *S*_w_ values of 9800 chemicals and was divided into three subsets: 6860 (70%) training set chemicals with their corresponding log *S*_w_ values, 1960 (20%) data points for the validation set, and a set of 980 (10%) chemicals for testing. To avoid a bias based on the selection of the training set we applied a fivefold split, varying the chemicals in training and validation set. We kept the initial test set for evaluation of each individual model. By generating tautomeric forms and different SMILES variants, the five different training sets were enlarged to 45,602 data points–46,835 data points for the training of the GNNs. We already applied this data augmentation strategy previously [24] and could demonstrate that the overall training was more stable and we achieved a better performance on the predictions. In this work, we reduced the number of tautomers to 50 per chemicals as we saw that there is no difference in the performance outcomes by this reduction.

The optimal GNN architecture for the corresponding datasets was determined by plotting the *rmse* values of the corresponding training and validation set over the epochs for different GNN setups and the five different data splits (SI1). The number of neurons per layer and the learning rate varied. Beforehand, different activation functions were tested. The optimal neural network was based on two hidden layers with 64 and 128 neurons. The learning rate was 0.0005, and the network was trained over 130 epochs. A pyramidal structure was tested as well, but did not perform as good as this variant (S1–5).

For fivefold cross-validation, we prepared five random splits of the training and validation sets (ensuring only that each chemical was at least once part of the validation set) and checked the individual optimal GNN parameters for each of the different splits (SI1-6–13). We came up with the same parametrization for the different GNNs and performed the corresponding training of the GNNs. The *r*^2^ values ranged from 0.839 to 0.870 for the different splits, the *q*^2^ values [34] ranged from 0.837 to 0.870, and the *rmse* ranged from 0.74 to 0.85). There is a slight difference in the corresponding *rmse* values, which could be explained by the different composition of the corresponding validation set.

Additionally, we developed a consensus model, where we used the mean of the five different GNNs and determined the corresponding standard deviation. The results for the fivefold cross-validation are depicted in Table 1, the corresponding plots of the experimentally determined log *S*_w_ versus predicted log *S*_w_ for the five GNNs and the consensus GNN are provided in SI2.

**Table 1:** Results of the five models and the consensus model, with each model trained on a distinct training set

Model	Split	Validation set			Test set		
		*r*^2^	*q*^2^	*rmse*	*r*^2^	*q*^2^	*rmse*
GNN1	1	0.870	0.870	0.735	0.873	0.873	0.728
GNN2	2	0.846	0.842	0.809	0.886	0.882	0.701
GNN3	3	0.839	0.839	0.821	0.881	0.880	0.708
GNN4	4	0.846	0.837	0.846	0.874	0.868	0.741
GNN5	5	0.853	0.841	0.841	0.881	0.871	0.734
Consensus	All				0.901	0.896	0.657
Average		0.850 (± 0.011)	0.845 (± 0.012)	0.807 (± 0.040)	0.878 (± 0.005)	0.875 (± 0.005)	0.722 (± 0.015)

We further developed five models based on the initial dataset (without data curation), the corresponding stats for the five GNNs are given in SI3. The performance of the model improved slightly by the corrections done, with a slightly higher* r*^2^ (0.901 versus 0.897) and a slightly lower *rmse* (0.657 vs 0.664) for both consensus models. It should be noted that the corresponding test dataset used for comparison was the corrected one.

Further, we used the test set of 980 chemicals to evaluate the performance of our GNNs (Table 1). According to the *r*^2^ values (0.873–0.886), *q*^2^ values (0.868–0.882), and the *rmse* values (0.70–0.74) of the five different GNNs trained, there is no clear indication that there was a bias introduced by the selection of a specific constellation of training set and validation set. The *r*^2^ and *q*^2^ are close to each other, indicating that there is no bias associated with the model used for the external prediction.

The corresponding standard deviations (SD) of the five GNNs applied in the consensus model are given for the test set in Table 2. We grouped the SDs to evaluate the overall prediction quality and reliability. As can be seen from Table 2, the *rmse* of the corresponding subset of test set chemicals is 0.44 for a SD ≤ 0.1 and 0.56 for a SD ≤ 0.2, therefore we assume a high reliability of the predictions. The *rmse* increases (0.59) for the SD range of 0.2 < SD ≤ 0.3. Thus, we declared the corresponding predictions as good reliability. For the SD range between ( <)0.3 and 0.5 the *rmse* values were 0.75 and 0.87. We assigned a moderate reliability. For predictions with an SD > 0.5, we determined an *rmse* of 0.93 and suggested to declare that the predicted log *S*_w_ values for these chemicals are less reliable. Note that this might be interpreted as a subjective categorization.

**Table 2:** Evaluation of the corresponding standard deviations of the five different predictions done by the 5 GNNs and information on the suggested quality and reliability of the predictions

Range of SD*	*n*	*rmse*	Max. error	Suggestion
0–0.1	38	0.436	1.50	High quality/reliability
0.1–0.2	309	0.559	2.79	Good quality/reliability
0.2–0.3	317	0.593	3.23	Good quality/reliability
0.3–0.4	171	0.748	2.58	Moderate quality/reliability
0.4–0.5	91	0.867	2.72	Moderate quality/reliability
> 0.5	54	0.929	3.32	Low quality/reliability

^*^Note that the range is given as 0 < SD <  = 0.1 (e.g.)

We also included an approach to depict the structural similarity in the applicability domain. The method is based on Tanimoto distances to its k-nearest neighbors and the prediction reliability of the training set chemicals [32]. Based on the work of Sahigara et al. [33], we determined an optimal *k* of 12 for our approach (see SI4). We applied the applicability domain for our test set, covering 98.7% (967/980 chemicals). The *rmse* of the subset of chemicals within the applicability domain is 0.655, and the *rmse* outside the applicability domain is 0.771. We uploaded the script for the determination of the applicability domain and a script for further application of the model to new datasets to the GitHub repository.

For the consensus model, the determined squared correlation coefficient is 0.901. Further, the predictive squared correlation coefficient *q*^2^ is 0.896 and the *rmse* of the test set was 0.66 log units (Table 3) with a maximal negative error (*mne*) of − 3.32 and a maximal positive error (*mpe*) of 3.23. The 95th percentiles of the negative and positive errors (95% neg, 95% pos) were − 1.25 and 1.48, respectively (Table 3). Although standard deviations for the experimental data are given only in some cases (and are not included in the comprised dataset), it is estimated that 0.5 to 0.6 log units shall be expected as a standard deviation for experimental solubility values [9, 35]. Thus, it is likely that the corresponding error for the prediction of log *S*_w_ should be higher. To test our data augmentation strategy, we developed for the first split (training and validation set) GNNs, where we did not apply the data augmentation. We tested several versions of the GNNs with various neurons implemented in the two layers (16 or 32) and achieved a minimum *rmse* of 0.960 on the test set and a corresponding *r*^2^ of 0.778. Thus, applying the data augmentation strategy improves our predictive performance.

**Table 3:** Performance of our GNN and other prediction tools on the test set

Test set *n* = 980	Consensus GNN	EPI suite	OCHEM	ACD GALAS
Predictions possible for	980	934	980	980
*r*^2^	0.901	0.718	0.885	0.835
*q*^2^	0.896	0.549	0.882	0.829
***rmse***	0.657	1.386	0.703*	0.845
*bias*	0.123	0.260	0.027	− 0.063
*mne*	− 3.32	− 7.15	− 4.99	− 5.74
*mpe*	3.23	5.80	3.74	4.53
95% neg	− 1.25	− 2.81	− 1.51	− 1.73
95% pos	1.48	3.30	1.52	1.75
Subset ionizable chemicals *n* = 257				
Predictions possible for	257	238	257	257
*r*^2^	0.797	0.529	0.787	0.715
*q*^2^	0.795	0.075	0.771	0.622
***rmse***	0.730	1.570	0.772	0.991
*bias*	0.054	0.310	− 0.013	− 0.379
*mne*	− 2.72	− 5.12	− 4.99	− 5.58
*mpe*	2.31	5.80	3.20	2.76
95% neg	− 1.49	− 3.19	− 1.75	− 2.26
95% pos	1.96	2.57	1.57	2.19
Subset neutral chemicals *n* = 723				
Predictions possible for	723	696	723	723
*r*^2^	0.918	0.758	0.902	0.868
*q*^2^	0.913	0.625	0.899	0.864
***rmse***	0.630	1.319	0.677	0.787
*bias*	0.147	0.243	0.041	0.050
*mne*	− 3.32	− 7.15	− 4.00	− 5.74
*mpe*	3.23	5.38	3.74	4.53
95% neg	− 1.19	− 2.70	− 1.43	− 1.49
95% pos	1.46	3.30	1.54	1.70

The performance of the GNN compared to three other available prediction tools (EPI Suite, ACD GALAS and OCHEM) for the test set of 980 chemicals. The statistics for the subsets of neutral and ionizable chemicals are given below the statistics of the total test set

^*^Note that parts of the test set were implemented in the training set of the model

**Fig. 3:**
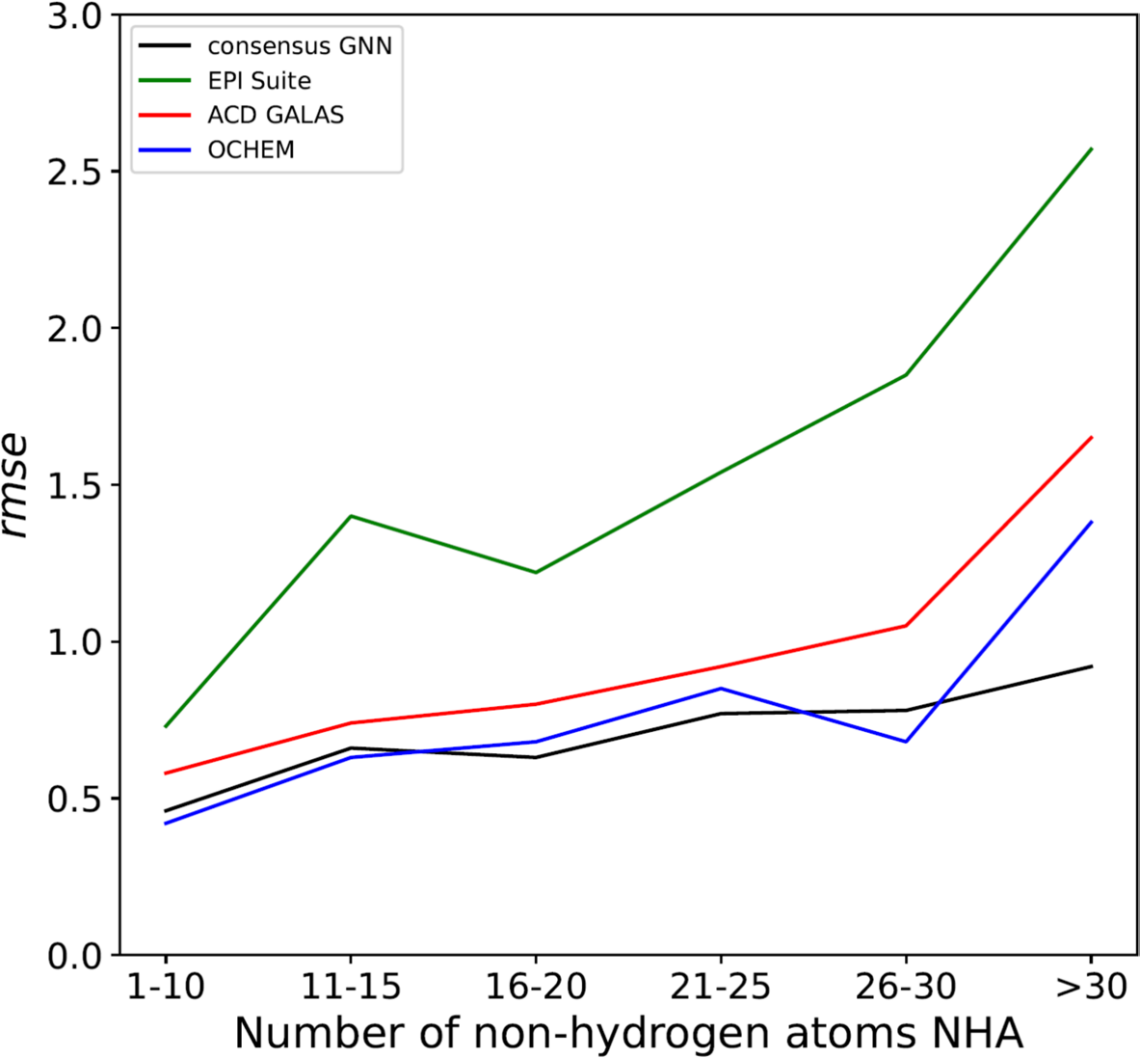
*Rmse* for different subgroups of the test set. The *rmse* of the test set is plotted versus the number of non-hydrogen atoms for our developed GNN and the three different prediction tools: EPI Suite, ACD GALAS, and OCHEM

We investigated the *rmse* for different subsets of the test set predictions based on our GNN and used the number of non-hydrogen atoms (NHAs) to characterize the different chemicals included in the test set (Fig. 3). As can be seen from Fig. 3, the lowest *rmse* value (0.46) is determined for the subset of chemicals with ≤ 10 NHAs (in total 225 chemicals), whereas *rmse* values of 0.66 and 0.63 are determined for chemicals with 11–15 NHAs (298 chemicals) and 16–20 NHAs (219 chemicals), respectively. The *rmse* value increases for chemicals with 21–25 NHAs (132 chemicals) to an *rmse* of 0.77 and an *rmse* of 0.78 for chemicals with 26–30 NHAs (51 chemicals). For large molecules with NHA > 30 (50 chemicals in the test set), the *rmse* is much higher, with an overall value of 0.92. One explanation for the higher *rmse* might be that the number of chemicals with experimental log *S*_w_ < − 6 is larger as compared to chemicals with log *S*_w_ > − 6 for this group (see SI5). Thus, the trend that higher *rmse* values are observed for the group of chemicals with NHA > 30 might be explained by the fact that these molecules are less soluble and the corresponding experimental error is larger as well (due to issues regarding the analytical determination like the limit of quantification of the corresponding method or the error in quantification of smaller concentrations related to the dilution of stock solutions).

### Comparison to other models

We compared the performance of our model to the developed consensus model AqSolPred of Sorkun et al. [25]. The authors used a subset of the AqSolDB (subset E, which is the log *S*_w_ collection of Huuskonen et al. [19]) as a test set to evaluate the performance of their models. The *rmse* of the consensus model AqSolPred for the E subset was 0.54. We applied our consensus GNN model to predict the log *S*_w_ values of the E subset (1291 log *S*_w_ data), resulting in a *rmse* of 0.43 (Table 4, GIT repository dataset.xlsx, predictions E dataset). Thus, our model seems to perform slightly better on this dataset. However, some data points of the E subset were implemented in our training sets as well and lead to this reduced *rmse*. So we re-trained the five models with reduced training sets and removed all data, which were implemented in the E subset for training. We again applied a consensus GNN with a corresponding *rmse* of 0.64 (Table 4). One explanation for the higher *rmse* value is that more chemicals are out of the corresponding applicability domain (STD > 0.5, reduced training sets 114 chemicals, initial training sets 69 chemicals).

**Table 4:** Performance of the consensus GNN for different solubility datasets commonly used in literature

Dataset from literature	Number of data points	Overlap of chemicals with our training data	Consensus GNN		Consensus GNN based on a reduced training set	
			*r*^2^	*rmse*	*r*^2^	*rmse*
Delaney	1128	966	0.950	0.488	0.889	0.711
Huuskonen*	1291	1125	0.960	0.427	0.905	0.637

^*^taken from AqSolDB—subset E

Further, we applied the consensus model to predict the Delaney subset used by Wu et al. [27] and Tang et al. [26]. Our model achieved an *rmse* of 0.49 on the subset (Table 4), which we extracted from Deepchem (1128 chemicals), the model of Wu et al. achieved an *rmse* of 0.58. Tang et al. used an increased dataset of 1311 chemicals for the comparison achieving an *rmse* of 0.66 (in comparison to Wu et al. *rmse* = 0.68). We again removed the corresponding chemicals of the dataset from our initial training sets and re-developed the models including the consensus GNN, by this, our *rmse* increased to 0.71 (Table 4). However, it is unclear how many datapoints of this set did overlap with the corresponding training sets of the methods applied in the previous studies.

Additionally, we compared experimental data with the predictions of our GNN model and those of three different software tools: EPI Suite [17], ACD Percepta (GALAS) [30], and OCHEM [29]. Only 934 SMILES codes of the test set chemicals could be processed in EPI Suite. The *rmse* of the test set predictions done by our consensus model was lowest (0.66 log units), the *rmse* of OCHEM was relatively close (0.70 log units). The corresponding *rmse* values of EPI Suite and ACD GALAS were higher, with 1.39 and 0.85 log units, respectively (Fig. 3, Table 3). Nevertheless, one needs to be aware that 547 chemicals of our test set were implemented in the training set of the OCHEM model. Thus, we selected the subset of chemicals not included in the training of the OCHEM model, and determined the corresponding *r*^2^, *q*^2^, and *rmse* for the remaining set of 433 chemicals for a better comparison of the performance. Our GNN achieved an *r*^2^ of 0.877, an *q*^2^ of 0.867, and a *rmse* of 0.700 on this subset. In comparison, the model implemented in OCHEM achieved an *r*^2^ of 0.788, an *q*^2^ of 0.768, and a *rmse* of 0.924 on this subset.

We also included the *rmse* values of the subsets of the test set for the different NHAs in Fig. 3. Especially for larger chemicals with NHAs > 30, the *rmse* is much higher than for smaller molecules. However, the *rmse* value for this subset was the lowest for the predictions of our GNN model (*rmse* = 1.05), followed by an *rmse* of 1.38 for the ACD GALAS model, an *rmse* of 1.65 for the model of OCHEM, and an *rmse* of 2.57 resulting from the predictions done by EPI Suite.

**Fig. 4:**
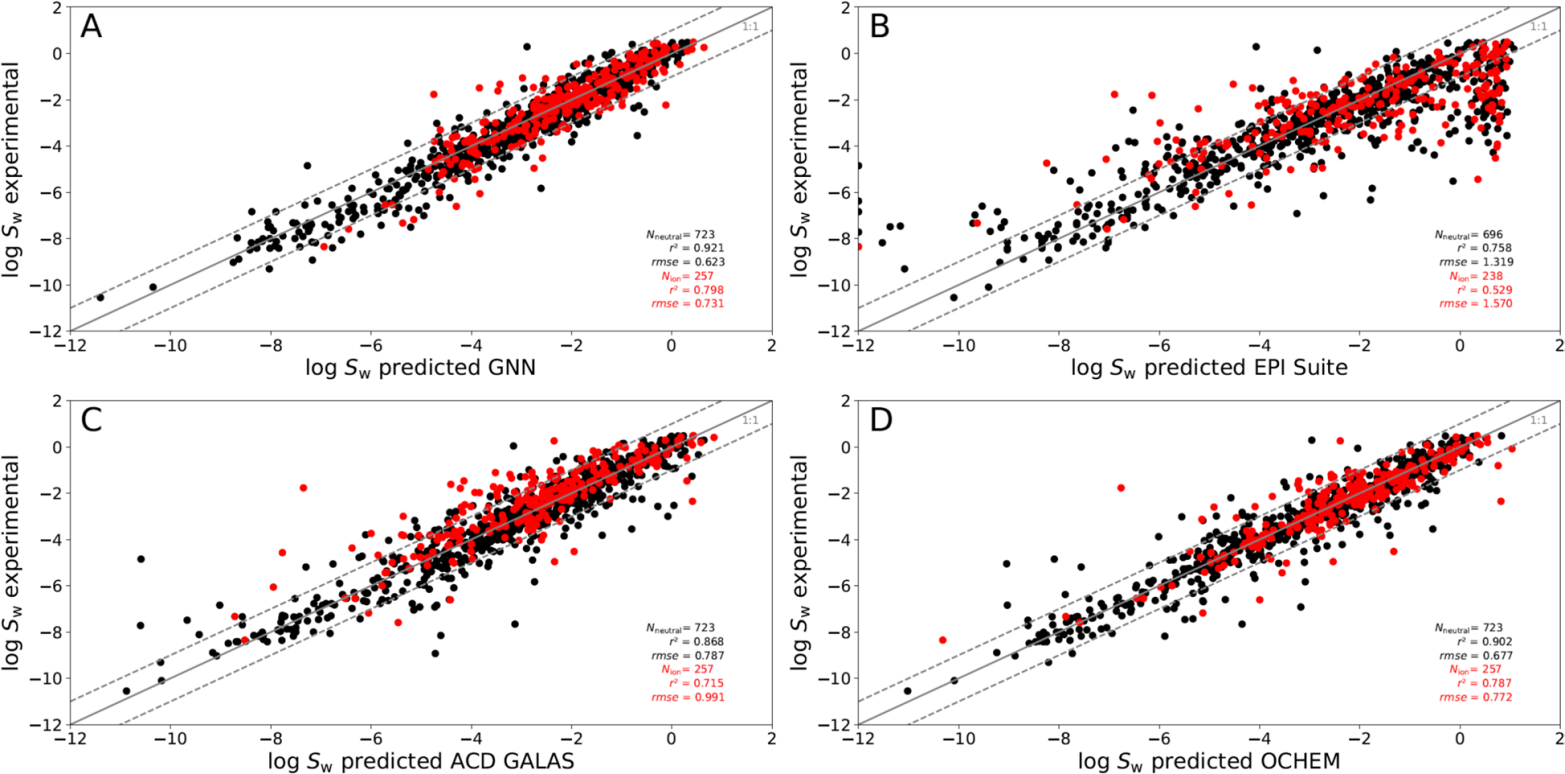
Comparison of the predictive outcomes of the developed GNN to other tools. Experimentally determined log *S*_w_ values are plotted against the predicted ones for **A** our developed GNN model, **B** EPI suite, **C** ACD GALAS, and **D** OCHEM. Potential ions are displayed in red, and neutral chemicals are shown in black

In many cases the pH value applied for the experimental determination of the log *S*_w_ value was not given. In addition, experimentally determined pKa values are not available for all the chemicals included in the dataset. Thus, we cannot ensure that only the intrinsic solubility is included in the dataset for each case. To cross-check whether ionizable chemicals can be identified as outliers, we marked them to identify specific patterns. As can be seen from Fig. 4, no outlier groups occur in the plots of experimentally determined log *S*_w_ values versus predicted ones. Outliers with more than one log unit difference to the experimentally determined log *S*_w_ value were checked individually. If we could not explicitly identify issues regarding the state of the chemical in the corresponding experiment, we did not exclude the corresponding value. We included the corresponding statistics for the subset of ionizable and neutral chemicals in Table 3.

**Fig. 5:**
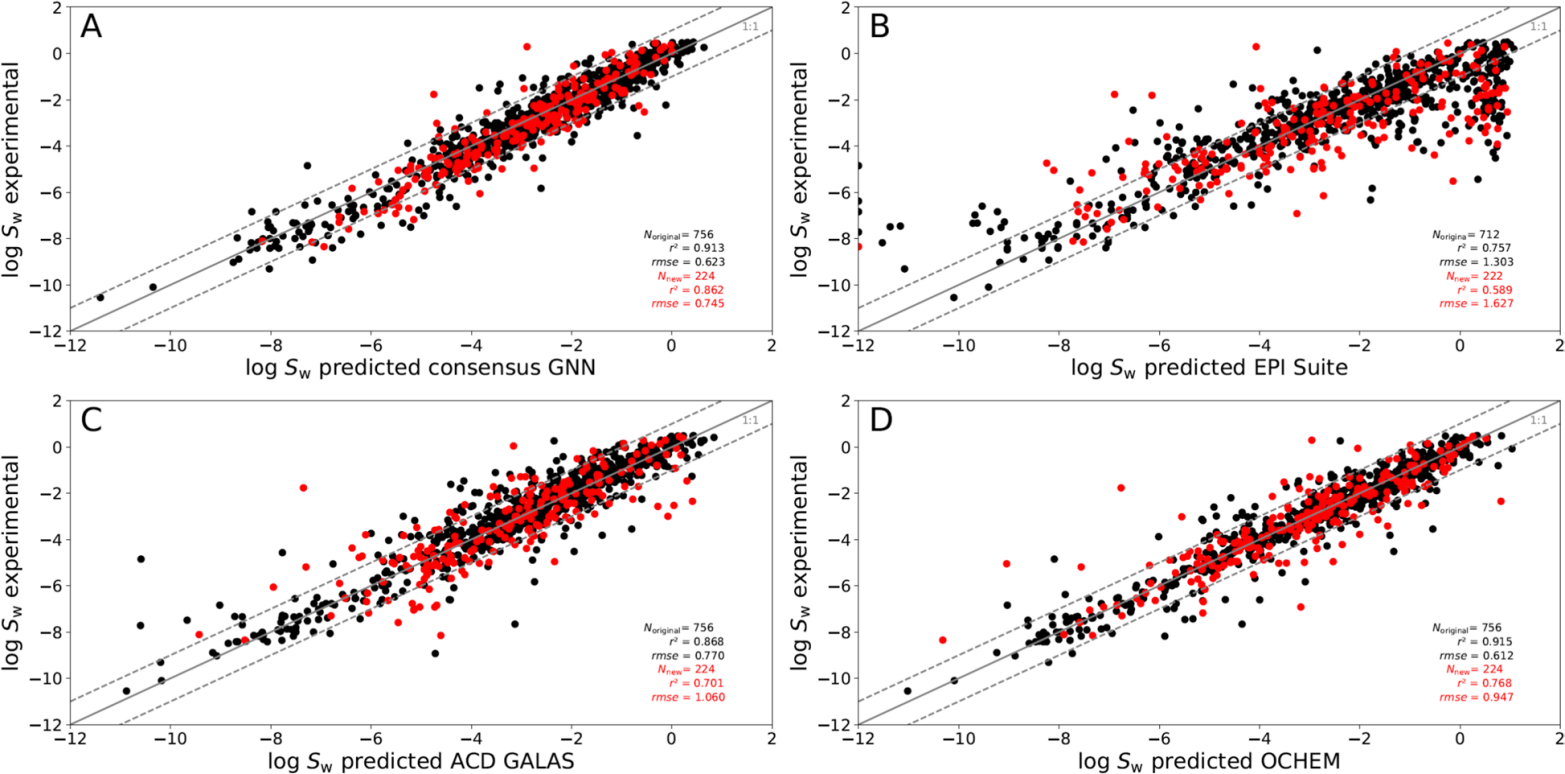
Comparison of the performance of the developed GNN for the test set split into original and novel data. Experimentally determined log *S*_w_ values are plotted against the predicted ones for **A** our developed GNN model, **B** EPI suite, **C** ACD GALAS, and **D** OCHEM. Data points of the original dataset are marked in black. New chemicals that were added to the original dataset are marked in red

We added a set of 2195 chemicals to the curated AqSolDB dataset (7605 chemicals) of Sorkun [15]. This new subset was randomly distributed into the subset used to generate the individual training and validation sets, and test set. For further comparison, we performed a statistical evaluation of the two subsets of the test set (Table 5, Fig. 5). As can be seen from Table 5, the *rmse* for the subset of novel data implemented in the test set is slightly higher for our developed consensus GNN (*rmse* = 0.74 new dataset, *rmse* = 0.63 original dataset). However, the differences between the *rmse* values of these subsets and those of the other tools applied are higher. For the predictions performed by OCHEM, the difference between the *rmse* of both subsets is even larger, with an *rmse* of 0.61 for the old subset and an *rmse* of 0.95 for the subset of the new chemicals included. The same trend can be observed for ACD GALAS with *rmse*s of 0.77 and 1.06 for the old and new subset and EPI Suite with *rmse*s of 1.30 and 1.63 for the old and new subset, respectively.

**Table 5:** Performance of our GNN and other prediction tools on the two different test subsets (original data and new data, which were added)

Test set *n* = 980	Consensus GNN	EPI suite	OCHEM	ACD GALAS
Subset original dataset *n* = 756				
Predictions possible for	756	712	756	756
*r*^2^	0.911	0.757	0.915	0.868
*q*^2^	0.908	0.620	0.913	0.863
rmse	0.630	1.303	0.612	0.770
*bias*	0.102	0.199	0.023	− 0.092
*mne*	− 2.58	− 7.15	− 3.25	− 5.74
*mpe*	3.23	5.80	3.31	4.53
95% neg	− 1.19	− 2.65	− 1.38	− 1.69
95% pos	1.45	3.14	1.31	1.50
Subset new dataset *n* = 224				
Predictions possible for	224	222	224	224
*r*^2^	0.862	0.589	0.768	0.701
*q*^2^	0.845	0.254	0.749	0.685
***rmse***	0.744	1.627	0.947	1.060
*bias*	0.191	0.456	0.040	0.037
*mne*	− 3.32	− 5.12	− 4.99	− 5.58
*mpe*	2.18	5.38	3.74	3.54
95% neg	− 1.37	− 3.65	− 2.37	− 2.01
95% pos	1.48	3.69	1.87	2.19

The performance of the GNN compared to three other available prediction tools (EPI Suite, ACD GALAS and OCHEM) for the test set of 980 chemicals. The statistics for the test subsets of original data and novel data are given

### Comparison of the model’s performance trained on a benchmark dataset

We additionally trained our model on the Delaney dataset (1128 datapoints) to allow for a direct comparison of the model’s performance to other models developed on the same dataset. As the number of datapoints is lower in comparison to our initial training set, we needed to repeat the hyperparameter optimization (see SI6 for details on the hyperparameters). We decided to randomly split the dataset into 80% for training and 10% for validation, and 10% for testing to allow for a better comparison to other models. We again applied a fivefold split to allow for a cross-validation approach. The GNN models developed achieved an average *r*^2^ of 0.852 (± 0.027) and *q*^2^ of 0.849 (± 0.028) on the validation sets with an *rmse* of 0.74 (± 0.09). The average *r*^2^, *q*^2^, and *rmse* values for the corresponding test sets are 0.894 (± 0.015), 0.888 (± 0.012), and 0.72 (± 0.06), respectively. In comparison, the three models developed by Cho and Choi [36] showed *rmse* values between 0.69 and 0.79 on a test set (10% of the dataset). The best performing model in their approach was also a graph convolutional neural network. The four NN models of Deng and Jia [37] were developed on a 80%/20% split and achieved *rmse* values from 0.97 to 1.05. Coley et al. [38] achieved an *rmse* of 0.56 (note that they used a reduced dataset of 1116 data points and a 80%/20% split). Wu et al. [27] achieved an *rmse* of 1.05 (validation set, 10%) and 0.97 (test set, 10%) with their GCNN model. The best performance was achieved by a message passing NN with *rmse* values of 0.55 and 0.58 for validation and test set, respectively. The same split was applied by Shen et al. [39], the corresponding test set *rmse* was at 0.58. Chen and Tseng could achieve an *rmse* of 0.56 based on a 90%/10% training set/test set split [40]. Especially message passing neural networks and multitask neural networks developed on this small dataset show better performances on the test sets. Nevertheless, we could demonstrate that the model’s performance is increased by training on the larger dataset.

## Conclusions

In this work, we developed a GNN to predict log *S*_w_ values for a broad spectrum of chemicals. We started by curating the AqSolDB and included log *S*_w_ values for 2,195 additional chemicals. Thus, our developed consensus GNN model with an *rmse* of 0.65 (for the independent test set) covers a broad spectrum of chemicals, which is demonstrated by the corresponding similarity-based approach to depict the applicability domain. Within our study, it became apparent that the overall performance and quality of the model’s prediction depends on the amount of data used for the training and on the quality of the input data. Therefore, it is essential that large datasets need to be curated. This is a time-consuming step that cannot be automatized by now, but it needs to be performed manually by independently checking each data point in literature or databases. Today, many studies show that deep learning models are trained on large datasets without data curation or pre-checks on consistency or plausibility, leading to poor performance and low-quality predictions. Further, there is a missing understanding of the underlying experiments and experimental errors, which is sometimes seen in overfitting in training these models.

## Supplementary Information

Supplementary Material 1.

## Data Availability

The dataset(s) supporting the conclusions of this article is available in the GIT repository nadinulrich/log_Sw_prediction: https://github.com/nadinulrich/log_Sw_prediction. The prediction tool will be implemented in our software PAULY.
