# Supplementary material for: Prediction of the water solubility by a graph convolutional-based neural network on a highly curated dataset
Source: J Cheminform. 2025 Apr 21;17:55. doi: 10.1186/s13321-025-01000-9 (PMC12012962; doi:10.1186/s13321-025-01000-9)
Supplement: Supplementary file 1 — Supplementary Material 1. [file 13321_2025_1000_MOESM1_ESM.pdf]

Supplementary Information - Prediction of the water solubility by a graph convolutional-based neural network on a highly curated dataset

Nadin Ulrich[1,2]*, Karsten Voigt[2], Anton Kudria[1], Alexander Böhme[1], Ralf-Uwe Ebert[1]

[1]Department of Exposure Science, Helmholtz Centre for Environmental Research – UFZ, Permoserstrasse 15, D-04318 Leipzig, Germany
[2]PAULY, Theresienstrasse 50, D-04129 Leipzig, Germany

*Corresponding Author: Department of Exposure Science, Helmholtz Centre for Environmental Research – UFZ, Permoserstrasse 15, D-04318 Leipzig, Germany

E-mail: nadin.ulrich@ufz.de

Table of content

SI1 Hyperparameter optimization of the neural networks 2

SI2 Correlations of experimentally determined and predicted log S_w values for the five different GNNs and the consensus GNN 15

SI 3 Comparison of the performance of models trained on the initial data set vs. models trained on the corrected dataset 16

SI4 Additional information on the similarity-based applicability domain 18

SI5 Detailed outlier analysis of the test set 20

SI6 Details on the training of the GNN on the Delaney dataset 23

SI1 Hyperparameter optimization of the neural networks

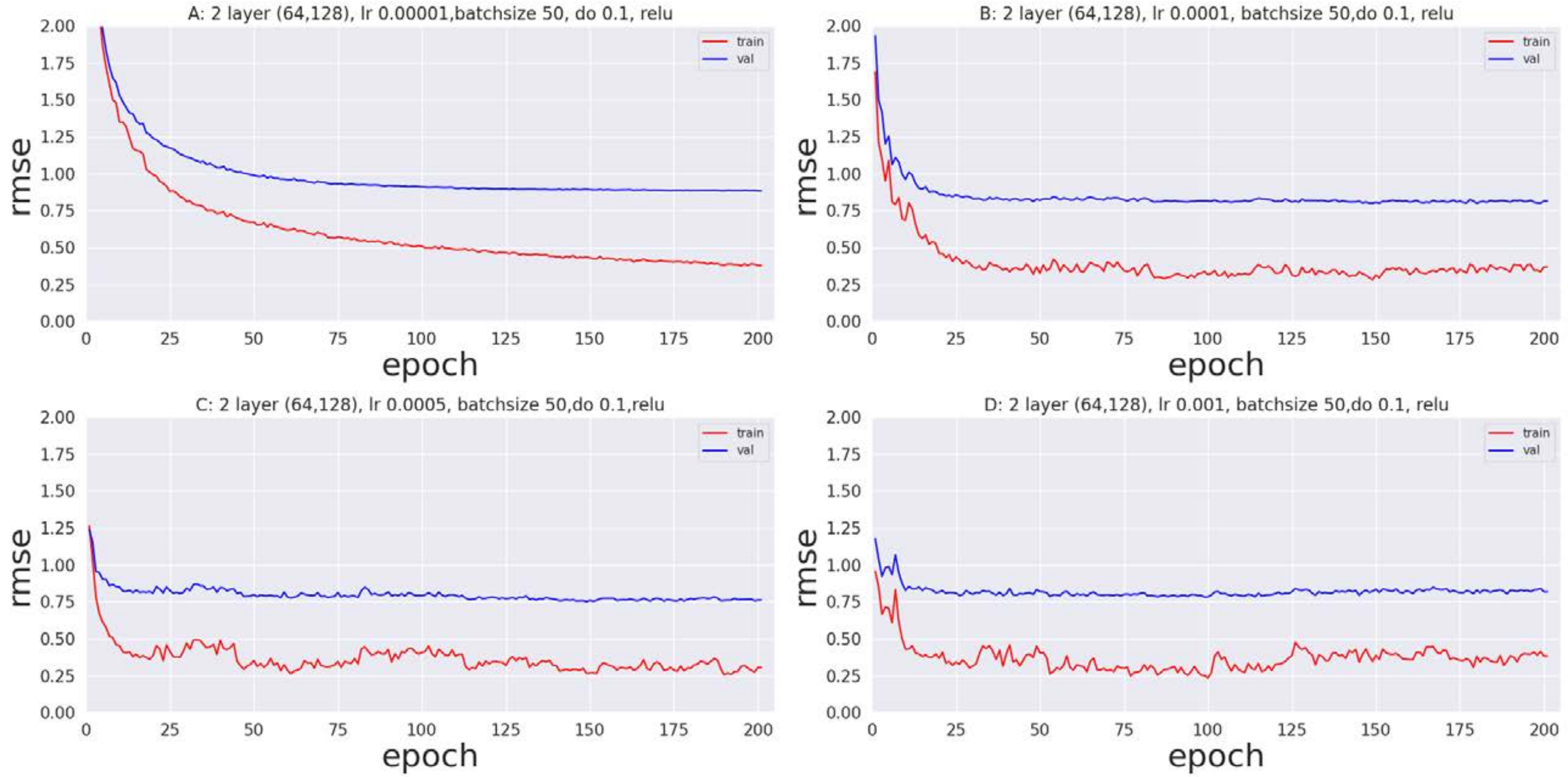

Figure S1-1. The *rmse* values of validation and training set (set 1) are plotted over the number of epochs for neural nets with different configuration. Here, neural nets with two layers including 64 and 128 neurons, respectively are shown, as activation function a relu function is used, the batch size is 50 and the dropout 0.1. Different learning rates are shown: (A) 0.0001, (B) 0.0001, (C) 0.0005, and (D) 0.001.

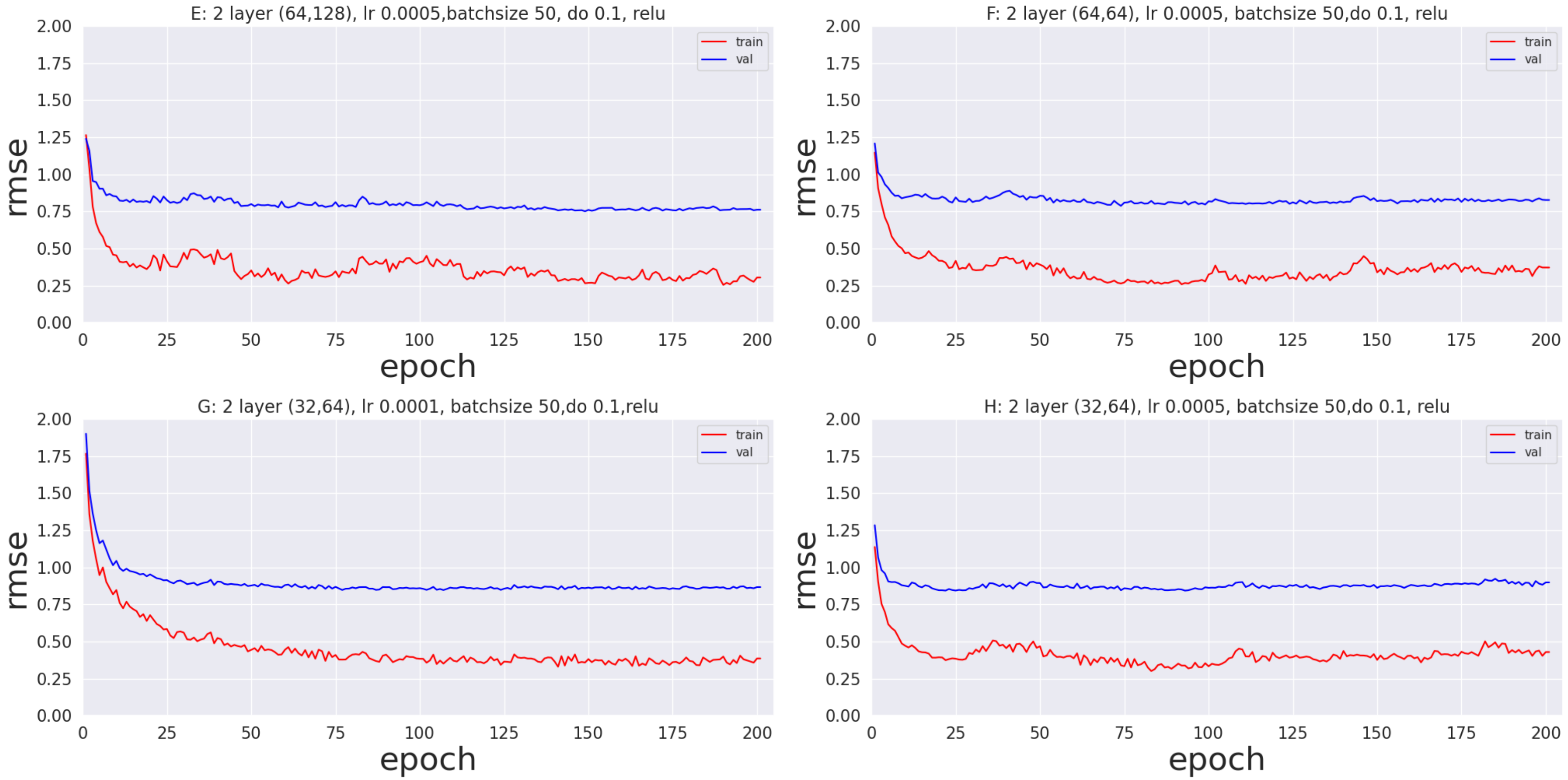

Figure S1-2. The *rmse* values of validation and training set (set 1) are plotted over the number of epochs for neural nets with different configuration. Here, neural nets with two layers including a different number of neurons are shown. As activation function a relu function is used, the batch size is 50 and the dropout 0.1, and the learning rate is 0.0001 (E-G) or 0.0005 (H). The neurons in the hidden layers are: (E) 64, 128, (F) 64,64, (G) and (H) 32,64.

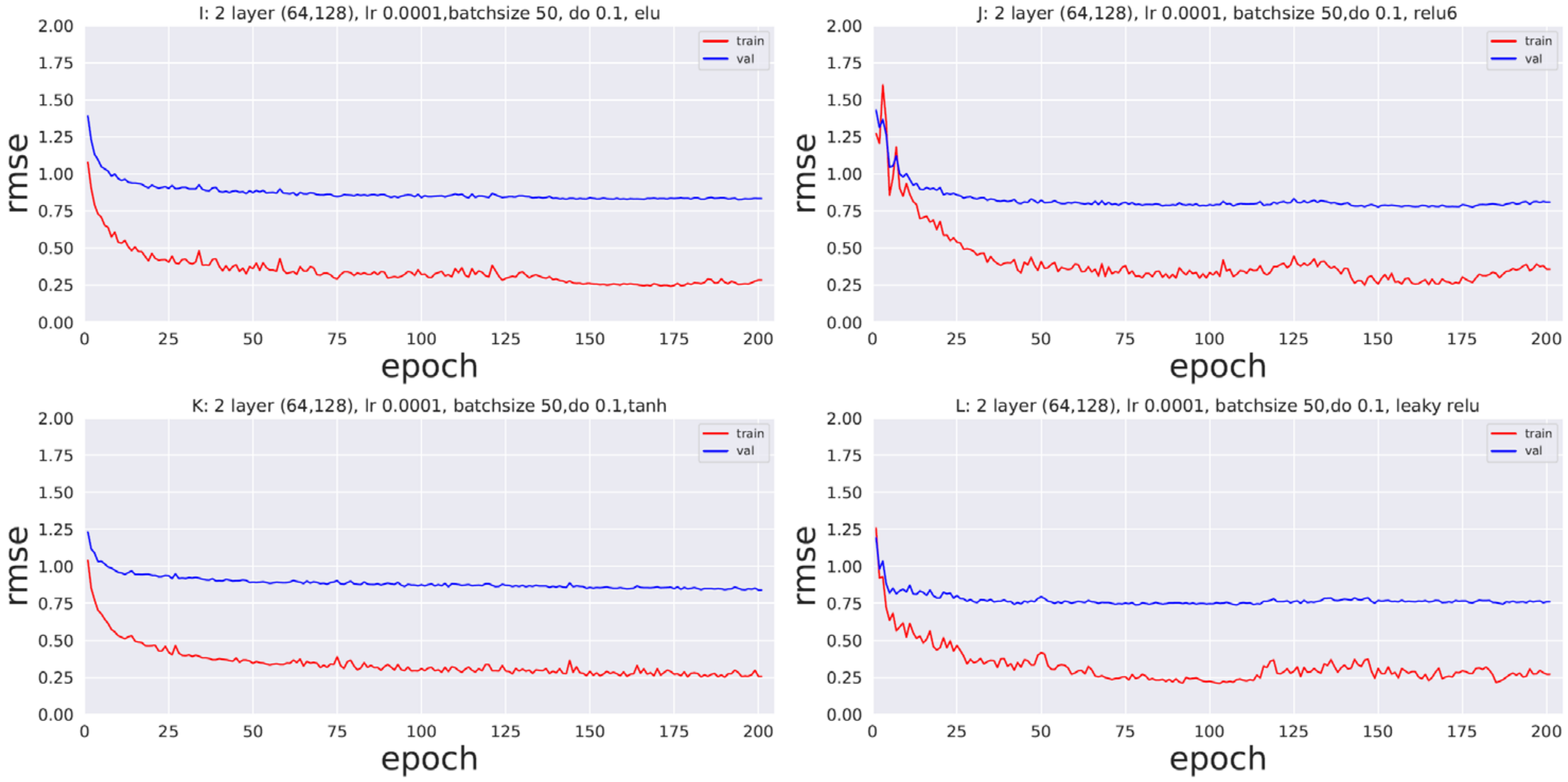

Figure S1-3. The *rmse* values of validation and training set (set 1) are plotted over the number of epochs for neural nets with different configuration. Here, neural nets with two layers including 64 and 128 neurons, respectively are shown, the batch size is 50 and the dropout 0.1, the learning rate is 0.0001. Different activation functions are shown: (I) elu, (J) relu6, (K) tanh, and (L) leaky relu.

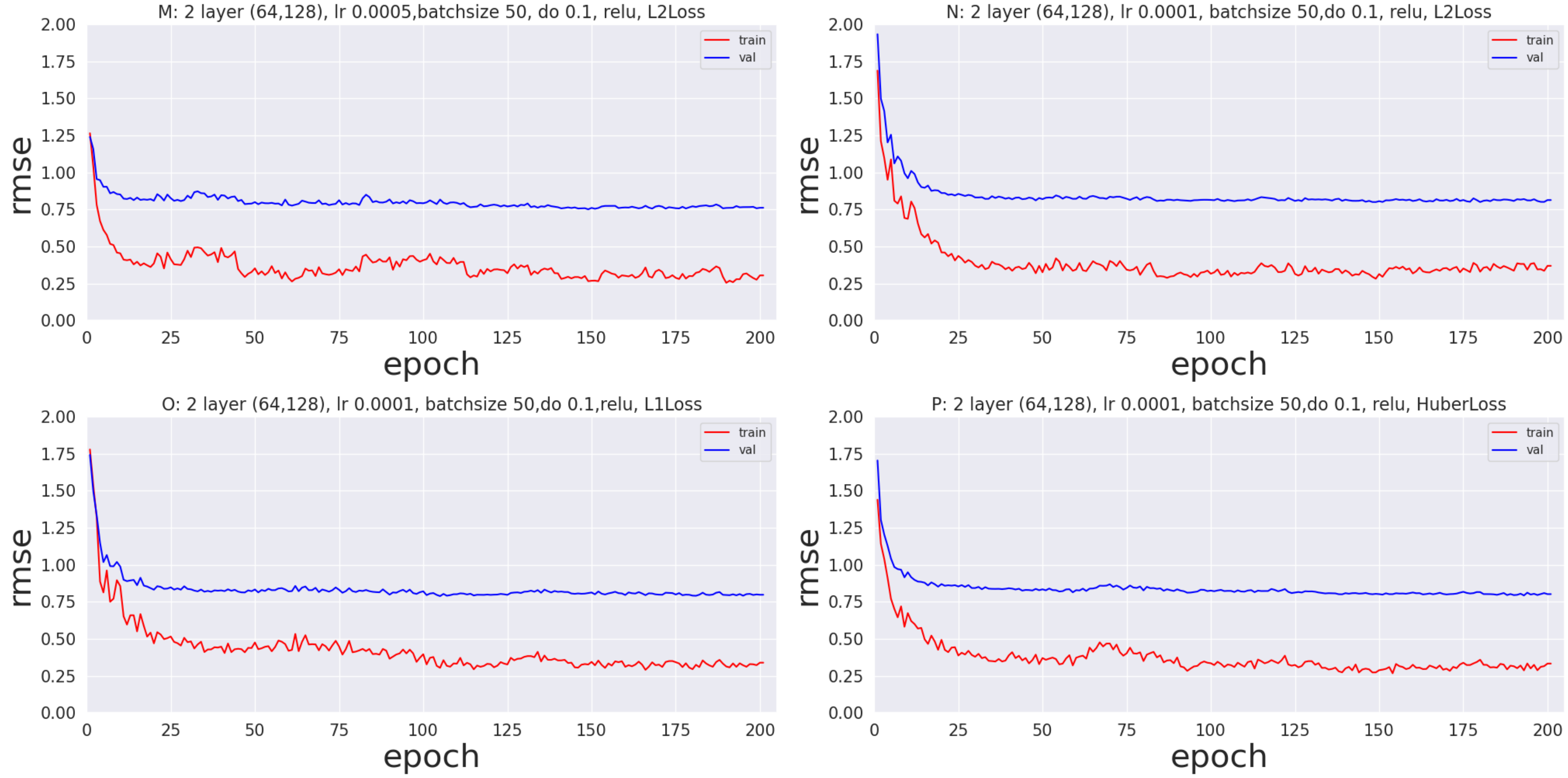

Figure S1-4. The *rmse* values of validation and training set (set 1) are plotted over the number of epochs for neural nets with different configuration. Here, neural nets with two layers including 64 and 128 neurons, respectively are shown, the batch size is 50 and the dropout 0.1, the learning rate is 0.0005 (M) or 0.0001 (N-P). Different loss functions are shown: (M, N) L2Loss (O) L1Loss, and (P) HuberLoss.

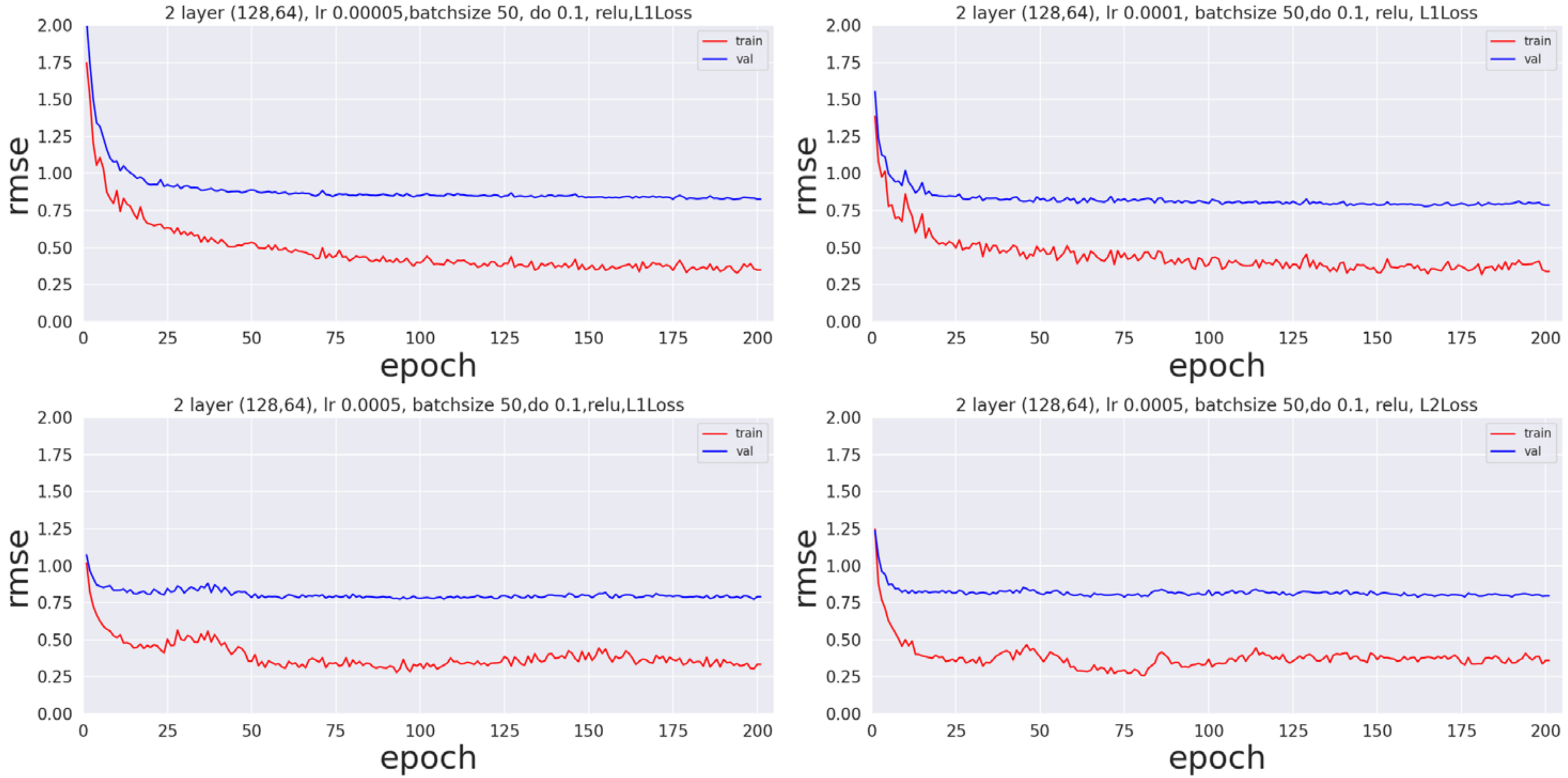

Figure S1-5. The *rmse* values of validation and training set (set 1) are plotted over the number of epochs for neural nets with different configuration. Here, neural nets with two layers including 128 and 64 neurons, respectively are shown, as activation function a relu function is used, a L1Loss function was applied, the batch size is 50 and the dropout 0.1. Different learning rates are shown: (A) 0.00005, (B) 0.0001, (C) 0.0005, and (D) 0.0005 (L2Loss).

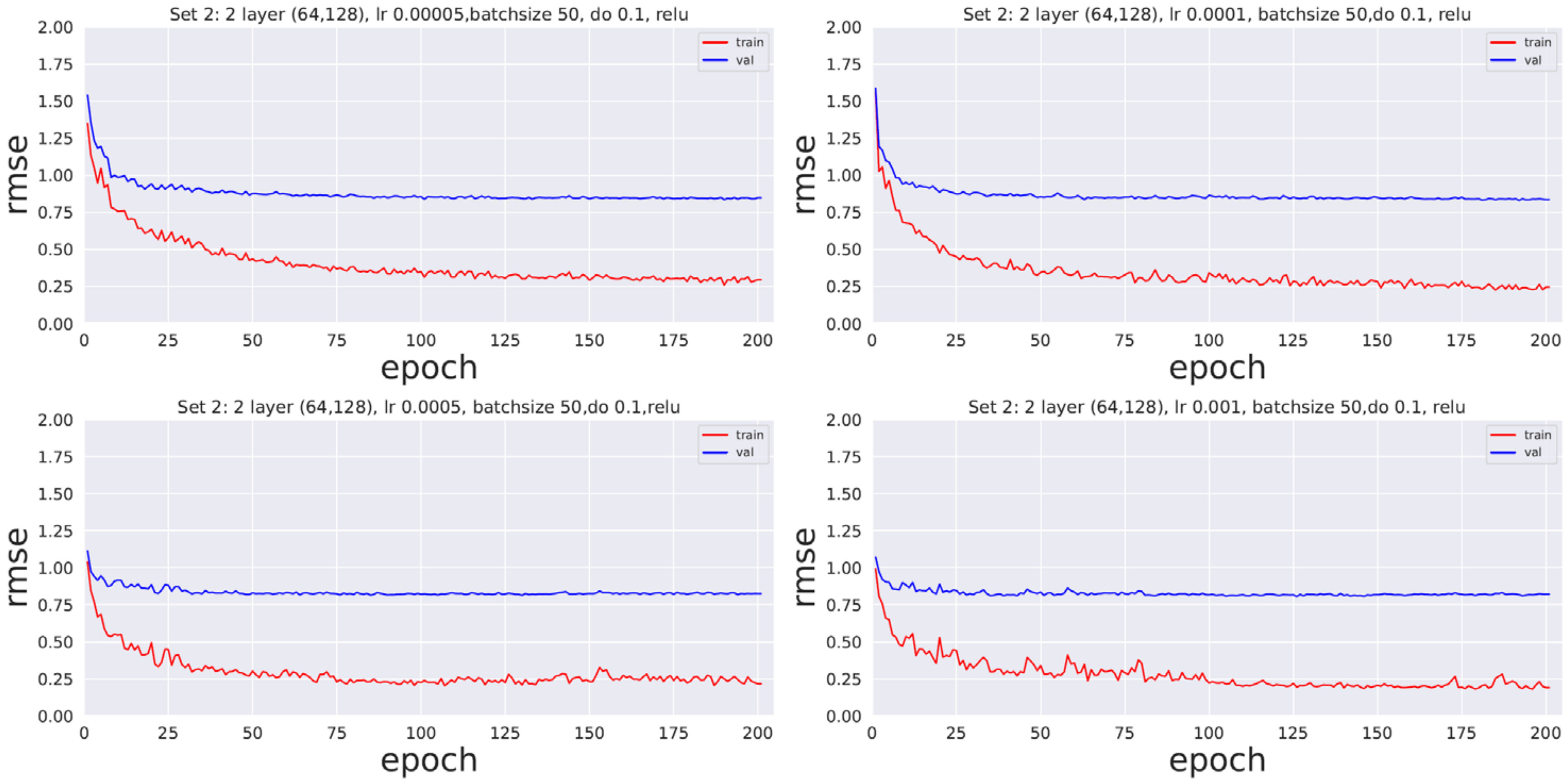

Figure S1-6. The *rmse* values of validation and training set (set 2) are plotted over the number of epochs for neural nets with different configuration. Here, neural nets with two layers including 64 and 128 neurons, respectively are shown, as activation function a leaky relu function is used, the batch size is 50 and the dropout 0.1. Different learning rates are shown: (A) 0.00005, (B) 0.0001, (C) 0.0005, and (D) 0.001.

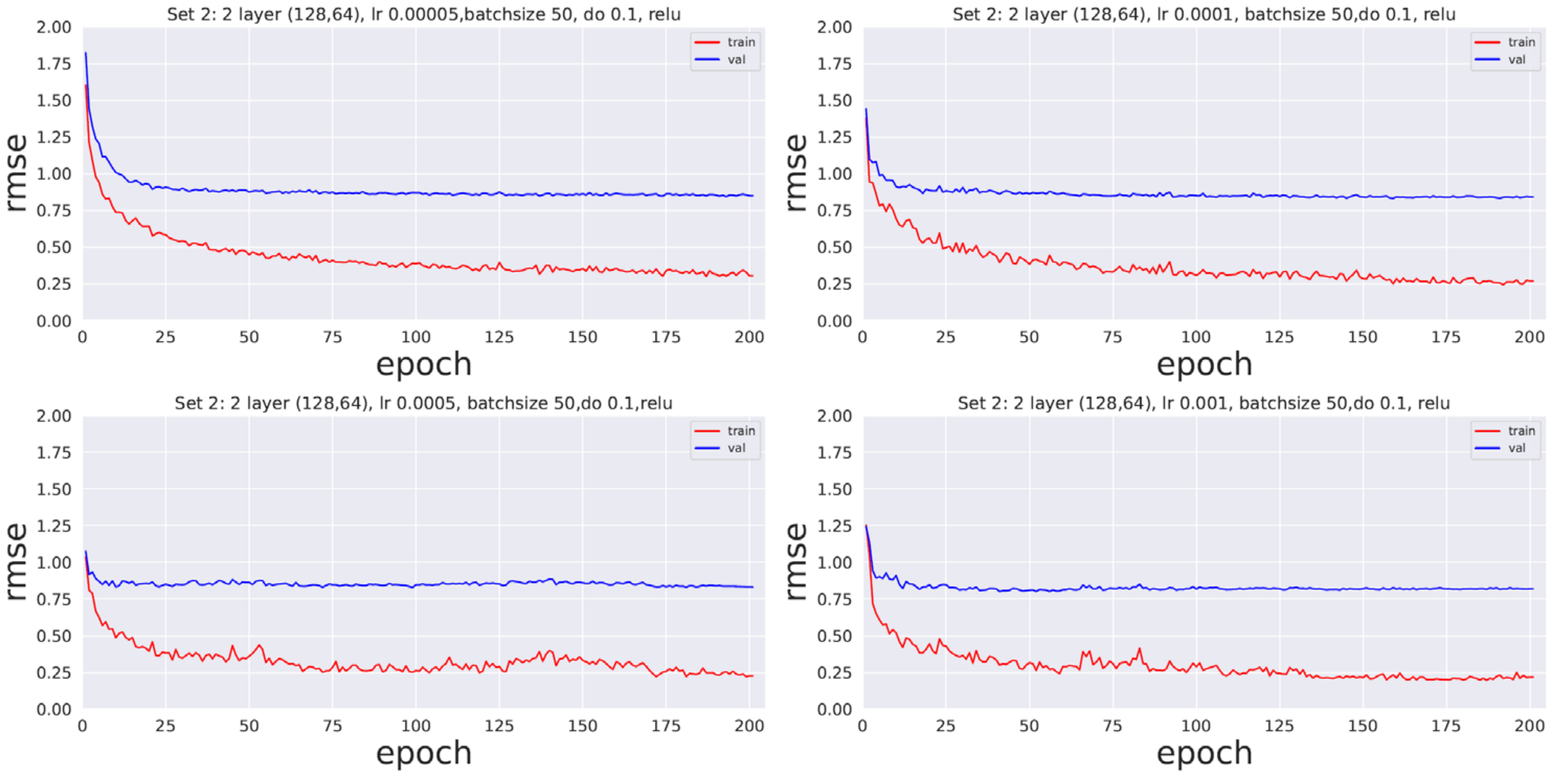

Figure S1-7. The *rmse* values of validation and training set (set 2) are plotted over the number of epochs for neural nets with different configuration. Here, neural nets with two layers including 128 and 64 neurons, respectively are shown, as activation function a leaky relu function is used, the batch size is 50 and the dropout 0.1. Different learning rates are shown: (A) 0.00005, (B) 0.0001, (C) 0.0005, and (D) 0.001.

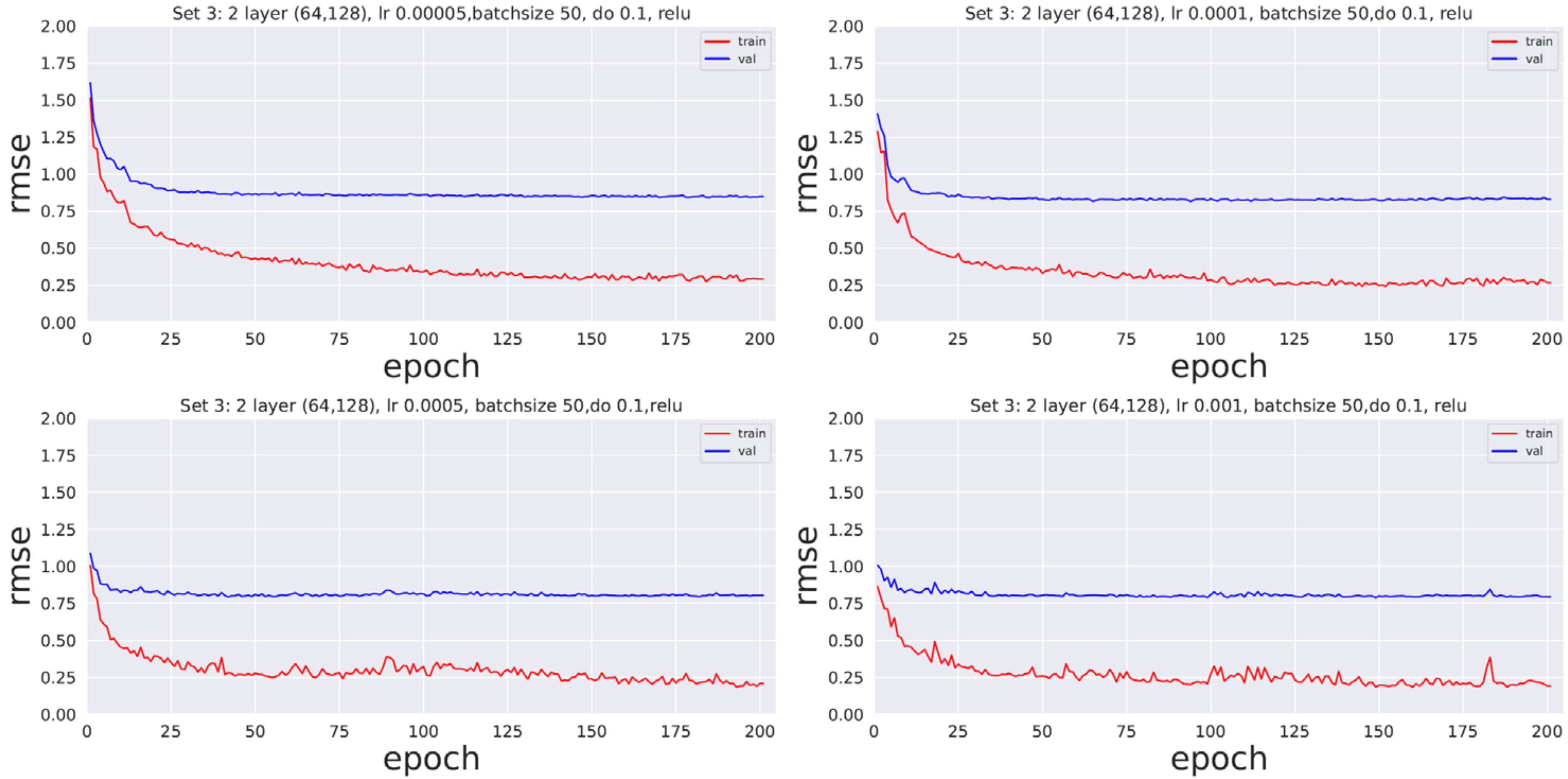

Figure S1-8. The *rmse* values of validation and training set (set 3) are plotted over the number of epochs for neural nets with different configuration. Here, neural nets with two layers including 64 and 128 neurons, respectively are shown, as activation function a leaky relu function is used, the batch size is 50 and the dropout 0.1. Different learning rates are shown: (A) 0.00005, (B) 0.0001, (C) 0.0005, and (D) 0.001.

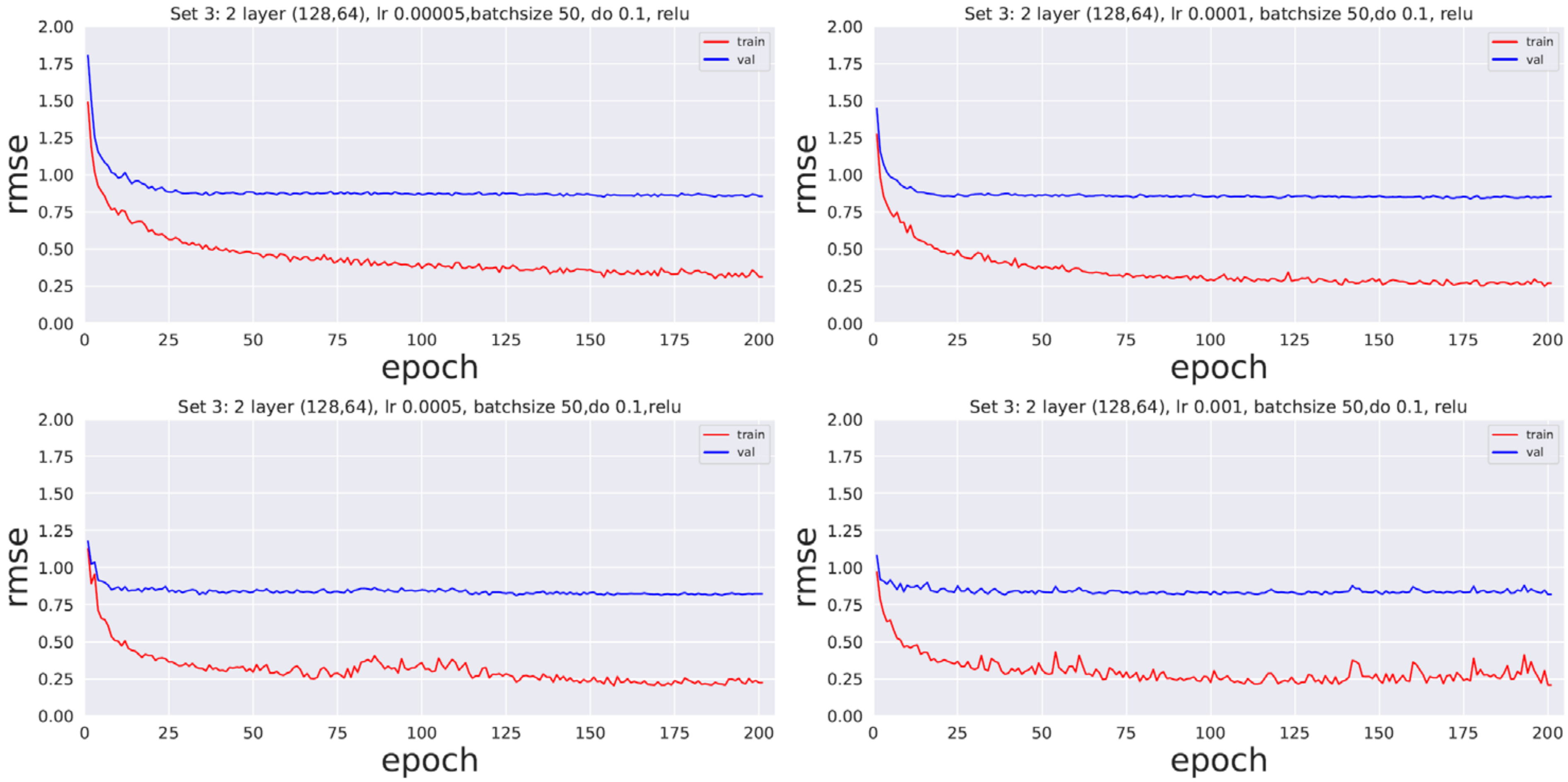

Figure S1-9. The *rmse* values of validation and training set (set 3) are plotted over the number of epochs for neural nets with different configuration. Here, neural nets with two layers including 128 and 64 neurons, respectively are shown, as activation function a leaky relu function is used, the batch size is 50 and the dropout 0.1. Different learning rates are shown: (A) 0.00005, (B) 0.0001, (C) 0.0005, and (D) 0.001.

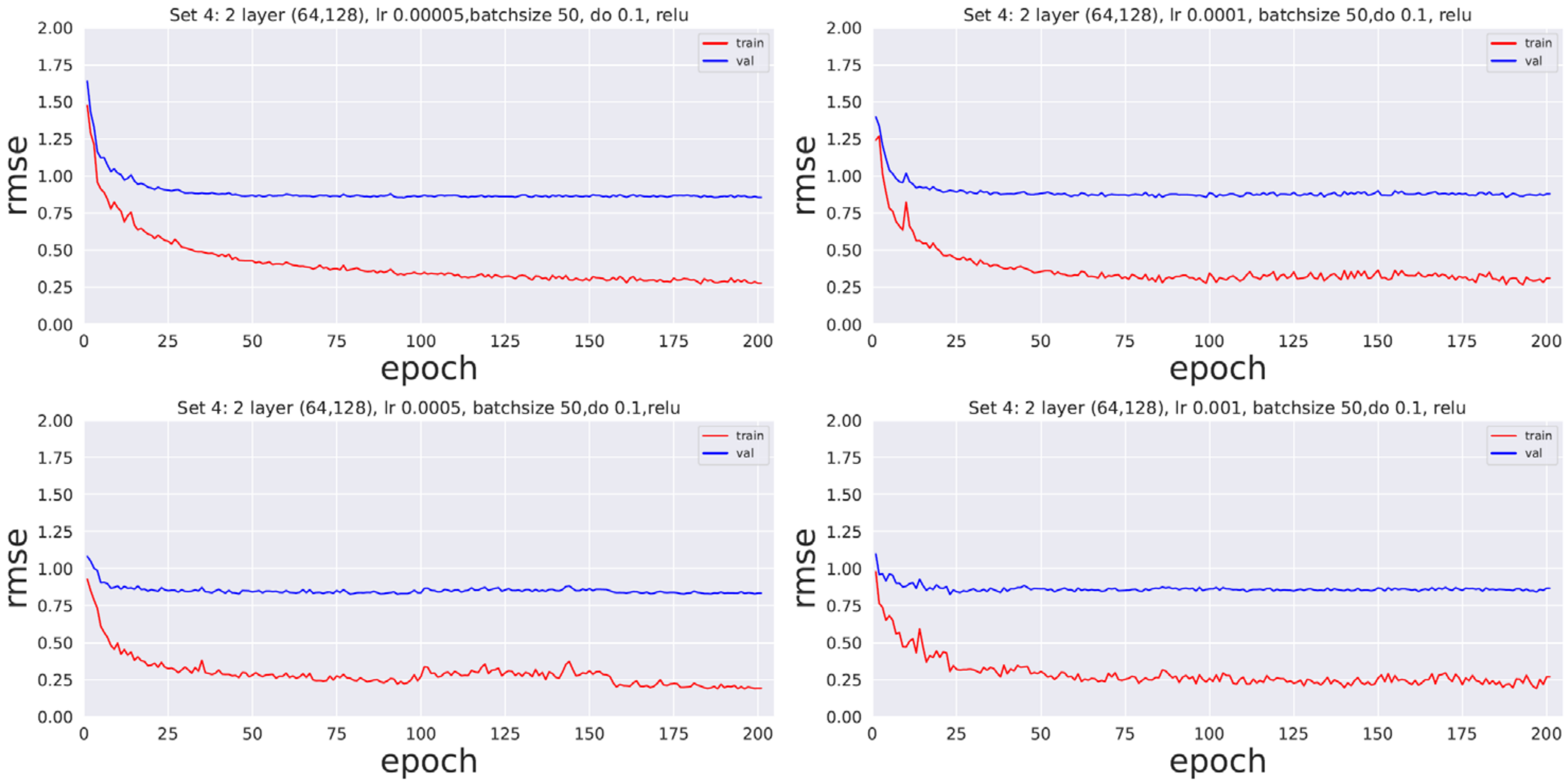

Figure S1-10. The *rmse* values of validation and training set (set 4) are plotted over the number of epochs for neural nets with different configuration. Here, neural nets with two layers including 64 and 128 neurons, respectively are shown, as activation function a leaky relu function is used, the batch size is 50 and the dropout 0.1. Different learning rates are shown: (A) 0.00005, (B) 0.0001, (C) 0.0005, and (D) 0.001.

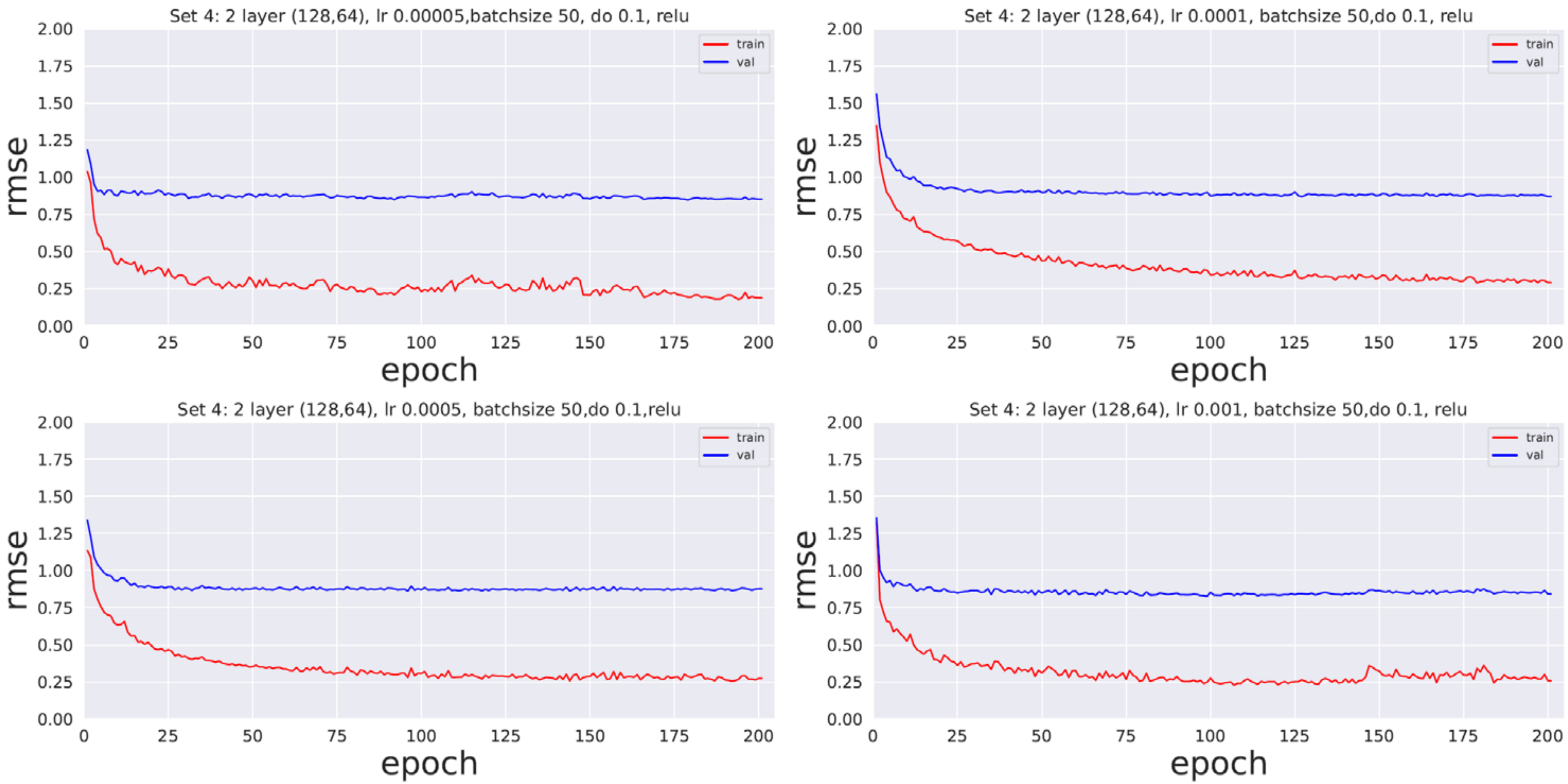

Figure S1-11. The *rmse* values of validation and training set (set 4) are plotted over the number of epochs for neural nets with different configuration. Here, neural nets with two layers including 128 and 64 neurons, respectively are shown, as activation function a leaky relu function is used, the batch size is 50 and the dropout 0.1. Different learning rates are shown: (A) 0.00005, (B) 0.0001, (C) 0.0005, and (D) 0.001.

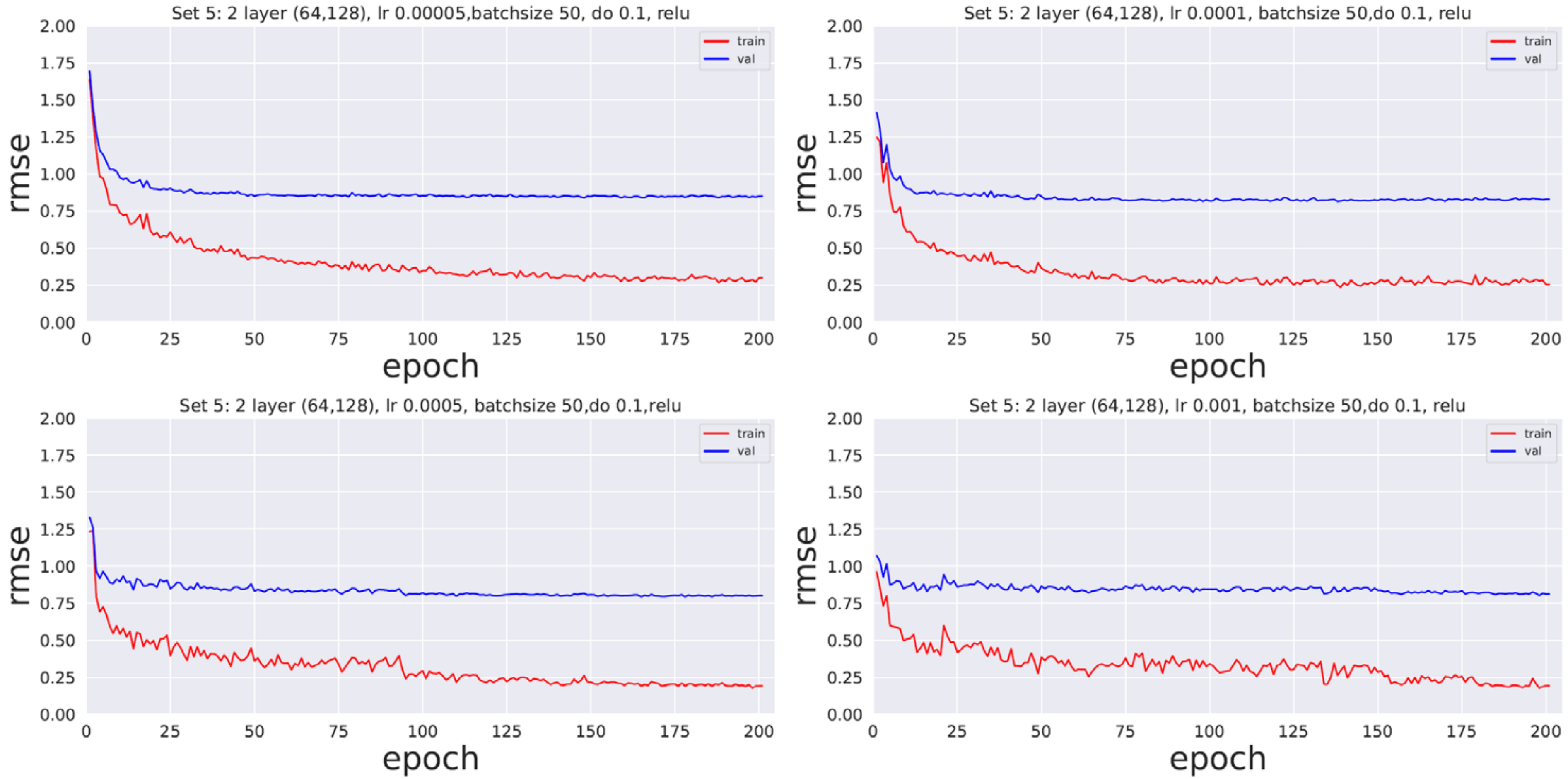

Figure S1-12. The *rmse* values of validation and training set (set 5) are plotted over the number of epochs for neural nets with different configuration. Here, neural nets with two layers including 64 and 128 neurons, respectively are shown, as activation function a leaky relu function is used, the batch size is 50 and the dropout 0.1. Different learning rates are shown: (A) 0.00005, (B) 0.0001, (C) 0.0005, and (D) 0.001.

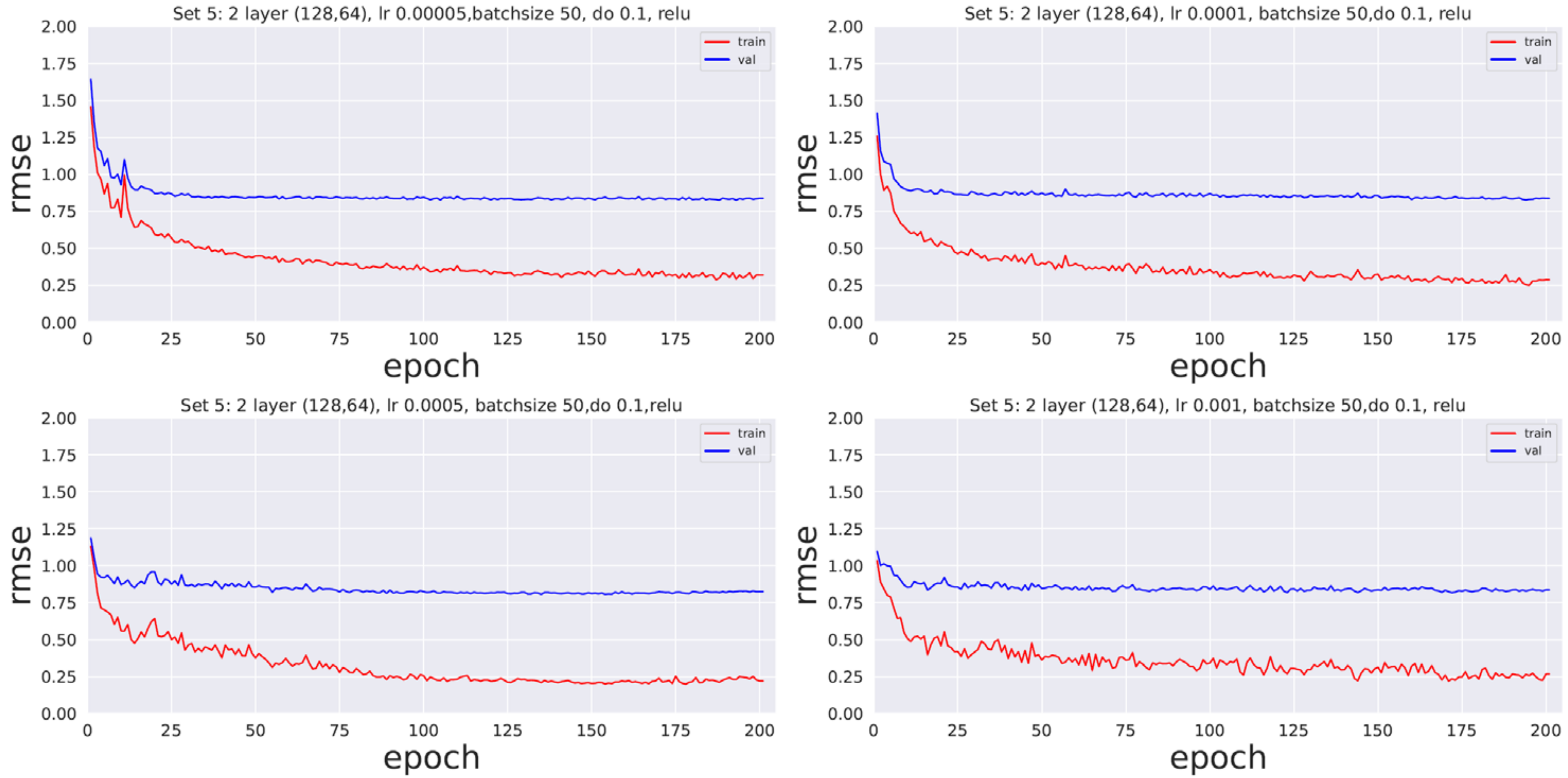

Figure S1-13. The *rmse* values of validation and training set (set 5) are plotted over the number of epochs for neural nets with different configuration. Here, neural nets with two layers including 128 and 64 neurons, respectively are shown, as activation function a leaky relu function is used, the batch size is 50 and the dropout 0.1. Different learning rates are shown: (A) 0.00005, (B) 0.0001, (C) 0.0005, and (D) 0.001.

SI2 Correlations of experimentally determined and predicted log S_w values for the five different GNNs and the consensus GNN

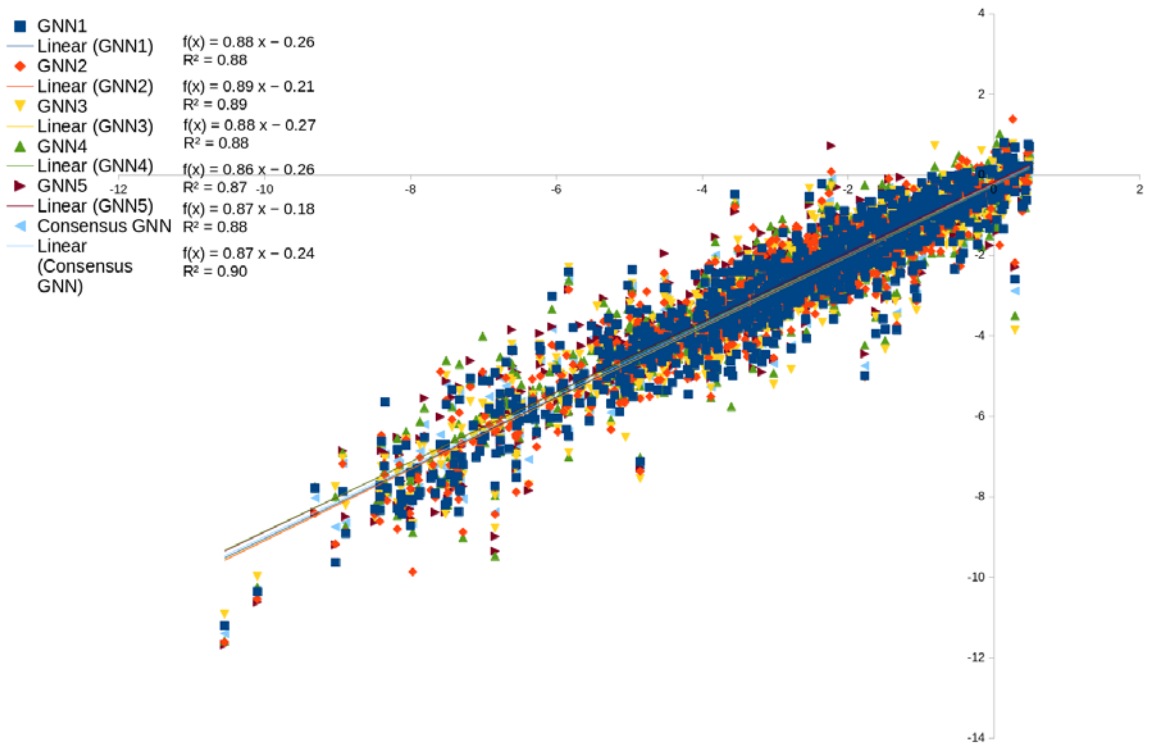

Figure S2-1. Correlations of experimentally determined and predicted log S_w values for the five different GNNs and the consensus GNN, linear fits with the corresponding equations are provided in the plot.

SI 3 Comparison of the performance of models trained on the initial data set vs. models trained on the corrected dataset

We checked whether our outlier curation procedure introduces a bias for the model and impacts the overall performance. We, therefore, used the initial dataset and proceeded with five splits of the validation and training set. We kept the test set as is but used the uncorrected version. As we had many exclusions, the number of chemicals in the different subsets of the initial dataset is higher (training set = 7242 chemicals, validation set = 2059 chemicals, test set = 1032 chemicals, note that inorganic chemicals, e.g., are still excluded and the cutoffs are introduced). To allow for a better comparison, we also applied the consensus GNN for the corrected, reduced test set (980 chemicals).

Table S3-1. Results of the five models, with each model trained on a distinct training set, for the validation set.

Model	Split	Uncorrected dataset						Corrected dataset					
		Validation set						Validation set					
		r^2	q^2	*rmse*	bias	mne	mpe	r^2	q^2	*rmse*	bias	mne	mpe
GNN1	1	0.843	0.842	0.830	0.055	-5.34	5.24	0.870	0.870	0.735	0.039	-3.04	3.82
GNN2	2	0.826	0.824	0.884	0.101	-5.90	5.77	0.846	0.842	0.809	0.118	-5.46	3.99
GNN3	3	0.831	0.825	0.874	0.140	-6.05	4.59	0.839	0.839	0.821	0.095	-4.98	3.82
GNN4	4	0.814	0.811	0.931	0.110	-5.72	5.43	0.846	0.837	0.846	0.198	-4.24	5.53
GNN5	5	0.830	0.822	0.886	0.188	-4.06	5.05	0.853	0.841	0.841	0.226	-3.89	4.49

Table S3-2. Test set results of the five models and the consensus model, each model trained on a distinct training set.

Model	Split	Uncorrected dataset, test set uncorrected						Uncorrected dataset, test set corrected						Corrected dataset, test set corrected					
		r^2	q^2	*rmse*	bias	mne	mpe	r^2	q^2	*rmse*	bias	mne	mpe	r^2	q^2	*rmse*	bias	mne	mpe
GNN1	1	0.863	0.862	0.778	0.047	-4.36	3.08	0.872	0.871	0.734	0.051	-4.07	3.50	0.873	0.873	0.728	0.045	-3.57	3.43
GNN2	2	0.871	0.869	0.760	0.089	-3.77	3.73	0.879	0.876	0.718	0.087	-3.21	3.31	0.886	0.882	0.701	0.123	-3.26	2.98
GNN3	3	0.870	0.868	0.763	0.107	-4.18	2.63	0.880	0.877	0.714	0.106	-3.34	2.97	0.881	0.880	0.708	0.082	-4.15	3.53
GNN4	4	0.867	0.865	0.770	0.076	-3.67	3.12	0.878	0.877	0.717	0.070	-3.67	3.04	0.874	0.868	0.741	0.155	-3.79	3.21
GNN5	5	0.862	0.855	0.797	0.174	-3.72	3.17	0.869	0.862	0.759	0.173	-3.70	3.68	0.881	0.871	0.734	0.208	-2.68	2.98
**Consensus**	**all**	**0.889**	**0.886**	**0.707**	**0.098**	**-3.43**	**2.91**	**0.897**	**0894**	**0.664**	**0.098**	**-3.43**	**3.30**	**0.901**	**0.896**	**0.657**	**0.123**	**-3.32**	**3.23**

SI4 Additional information on the similarity-based applicability domain

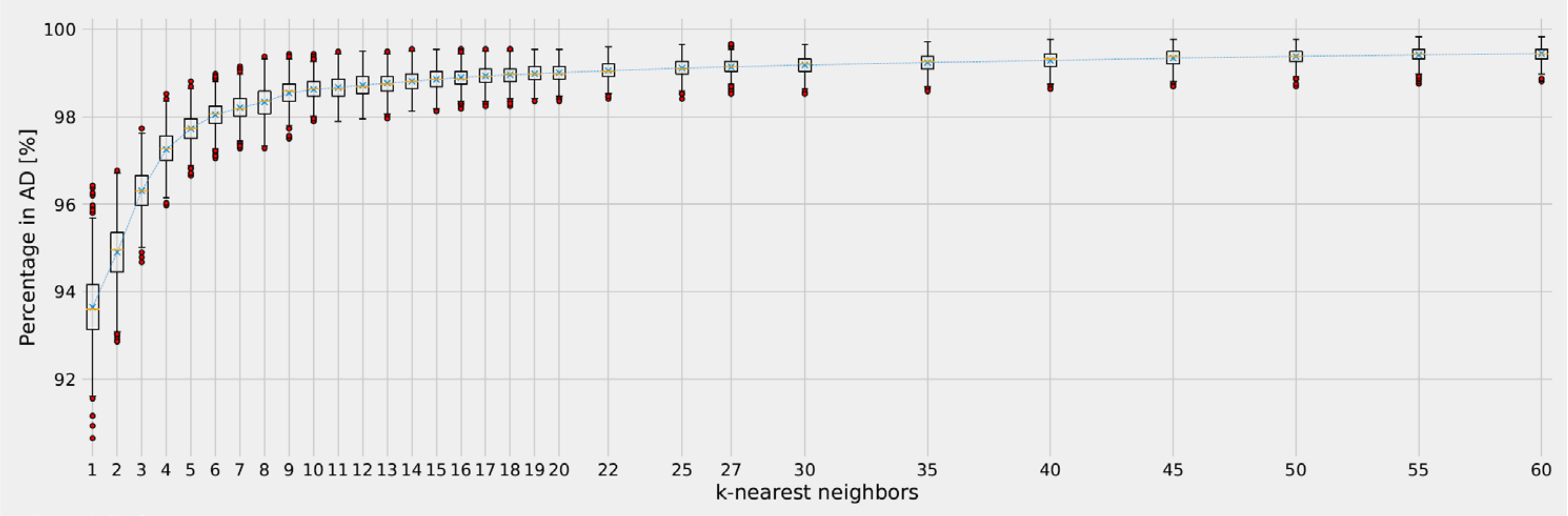

Figure S4-1. Distribution of the applicability domain inclusion percentage for the 1000 80/20 splits of the training set for different selected *k* values.

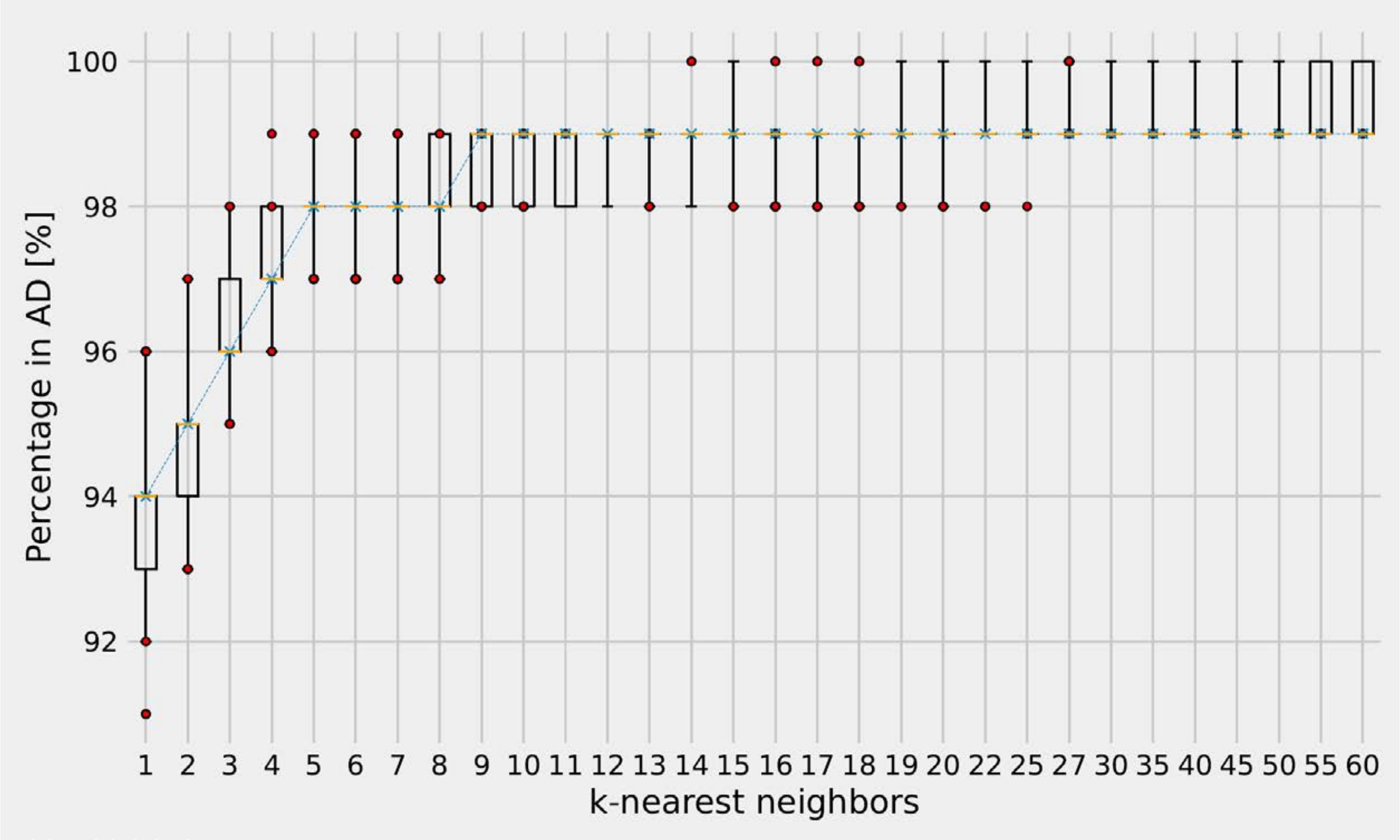

Figure S4-2. Distribution of the applicability domain inclusion percentage for the 1000 80/20 splits of the training set for different selected *k* values, note that the values are rounded to integers in this plot for better overview of the plot. Based on this plot, we selected a *k* of 12.

SI5 Detailed outlier analysis of the test set

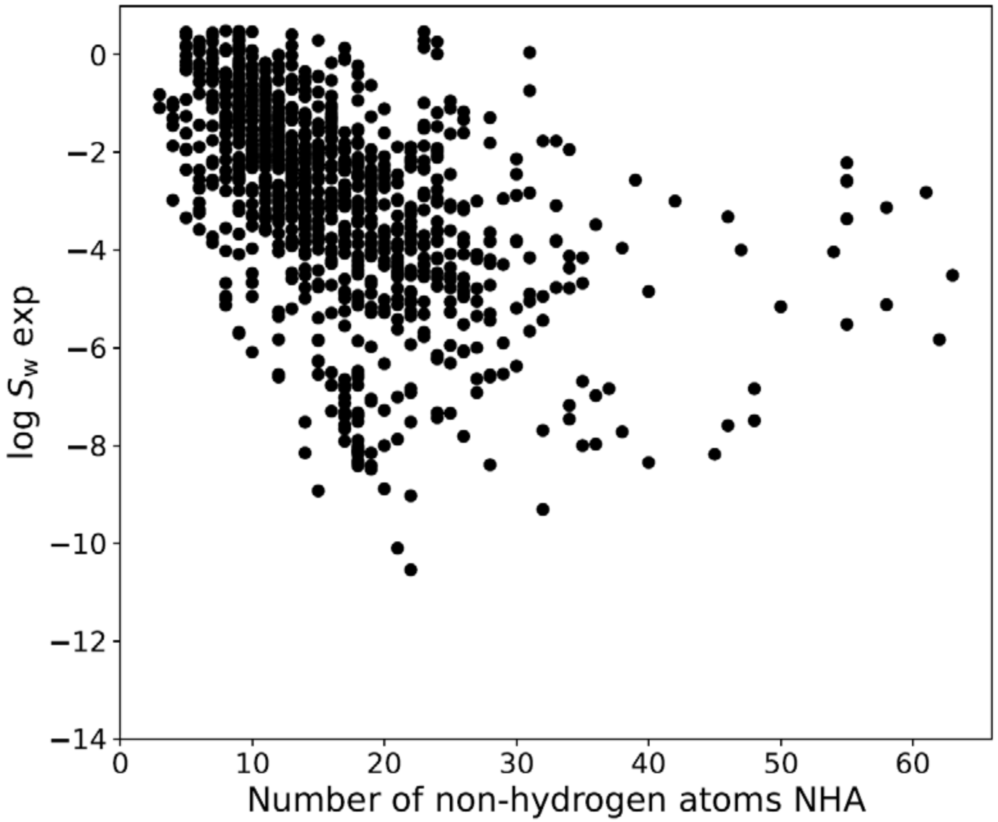

Figure S5-1. The experimental log S_w values of the test set are plotted over number of non-hydrogen atoms NHA. The number of chemicals with experimental log S_w values <-6 is larger in relation to the number of chemicals with experimental log S_w values >-6 for this group. Thus, the trend that greater *rmse* values are observed for the group of chemicals with NHA >30 might be explained by the fact that these molecules are less soluble and the corresponding experimental error is larger (due to issues regarding the limit of quantification).

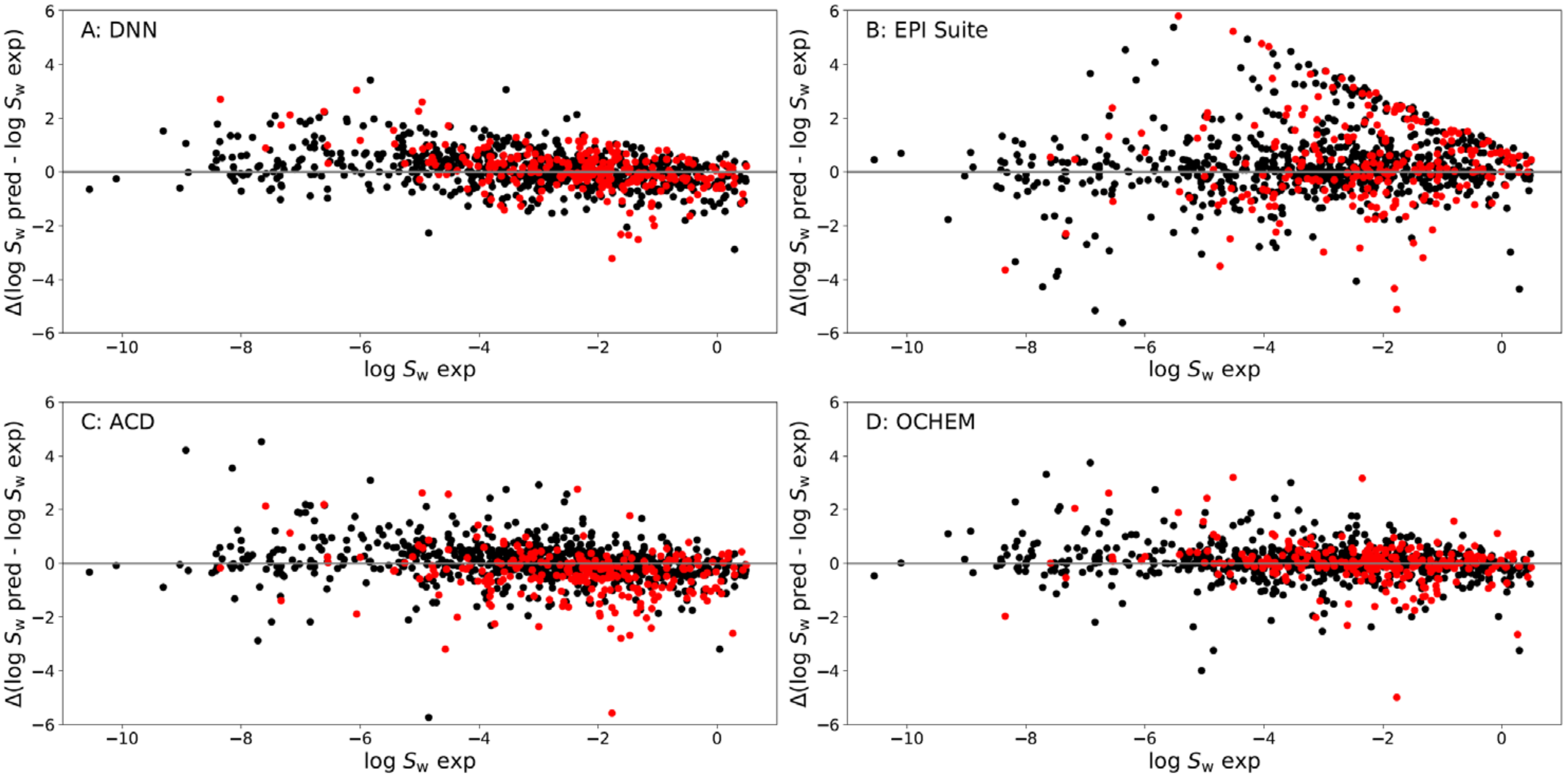

Figure S5-2. The differences between the predicted log S_w value and the corresponding experimental log S_w value are plotted over the experimental log S_w values for the test set chemicals. Neutral chemicals are marked in black, potential ions are marked in red. The following prediction tools are shown: A) DNN model, B) EPI Suite, C) ACD Galas, D) OCHEM.

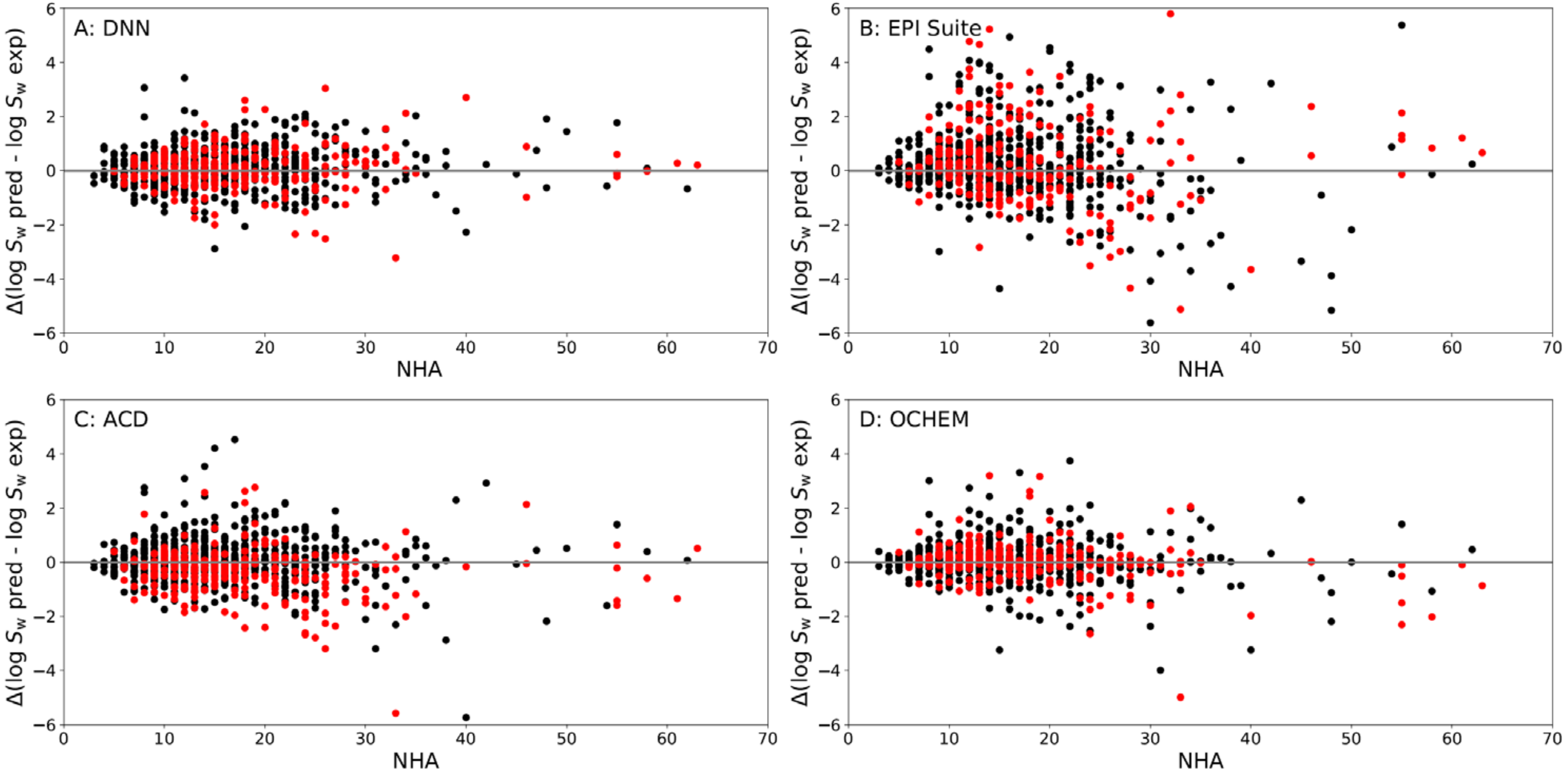

Figure S5-3. The differences between the predicted log S_w value and the corresponding experimental log S_w value are plotted over the number of nonhydrogen atoms NHA for the test set chemicals. Neutral chemicals are marked in black, potential ions are marked in red. The following prediction tools are shown: A) DNN model, B) EPI Suite, C) ACD Galas, D) OCHEM.

SI6 Details on the training of the GNN on the Delaney dataset

To allow for a comparison of the model's performance with other models, we trained our model on the Delaney dataset. This dataset contains 1,128 molecules with the corresponding solubility data and is commonly used for comparison of the performance of different models. As the dataset is relatively small, we first performed a hyperparameter optimization to avoid overparameterization. We ended up with a GNN containing two hidden layers, each including 32 neurons. The learning rate applied was 0.0005. We used a leaky ReLu as activation function and L1Loss function, and trained the model over 150 epochs.
